# A metabolomic study of the effect of *Candida albicans* glutamate dehydrogenase deletion on growth and morphogenesis

**DOI:** 10.1038/s41522-019-0086-5

**Published:** 2019-04-08

**Authors:** Ting-Li Han, Richard D. Cannon, Sandra M. Gallo, Silas G. Villas-Bôas

**Affiliations:** 1 School of Biological Sciences, The University of Auckland, Auckland, New Zealand; 2 Department of Obstetrics and Gynecology, The First Affiliated Hospital of Chongqing Medical University, Chongqing, China; 3 Sir John Walsh Research Institute, University of Otago Faculty of Dentistry, Dunedin, New Zealand

**Keywords:** Cellular microbiology, Antimicrobials

## Abstract

There are two glutamate dehydrogenases in the pathogenic fungus *Candida albicans*. One is an NAD^+^-dependent glutamate dehydrogenase (*GDH2*) and the other is an NADPH-dependent glutamate dehydrogenase (*GDH3*). These two enzymes are part of the nitrogen and nicotinate/nicotinamide metabolic pathways, which have been identified in our previous studies as potentially playing an important role in *C. albicans* morphogenesis. In this study, we created single gene knockout mutants of both dehydrogenases in order to investigate whether or not they affect the morphogenesis of *C. albicans*. The *GDH* genes were deleted and the phenotypes of the knockout mutants were studied by growth characterisation, metabolomics, isotope labelling experiments, and by quantifying cofactors under various hyphae-inducing conditions. We found that the *gdh2/gdh2* mutant was unable to grow on either arginine or proline as a sole carbon and nitrogen source. While the *gdh3*/*gdh3* mutant could grow on these carbon and nitrogen sources, the strain was locked in the yeast morphology in proline-containing medium. We detected different concentrations of ATP, NAD^+^, NADH, NAPD^+^, NADPH, as well as 62 other metabolites, and 19 isotopically labelled metabolites between the mutant and the wild-type strains. These differences were associated with 44 known metabolic pathways. It appears that the disequilibrium of cofactors in the *gdh3*/*gdh3* mutant leads to characteristic proline degradation in the central carbon metabolism. The analysis of the *gdh2*/*gdh2* and the *gdh3*/*gdh3* mutants confirmed our hypothesis that redox potential and nitrogen metabolism are related to filament formation and identified these metabolic pathways as potential drug targets to inhibit morphogenesis.

## Introduction

*Candida albicans* is a polymorphic fungus that grows on various human mucosal surfaces. The morphological switch from budding yeast to filamentous forms is often associated with its biological adaptation as an opportunistic pathogen of humans. The yeast-to-hyphal transition has been previously reported to play an important role in *C. albicans* systemic infections by enabling the hyphae to penetrate endothelial tissue and subsequently seed the bloodstream with yeast cells ^1–3^. *C. albicans* yeast cells are also capable of surviving during phagocytosis by triggering filamentous growth and eventually bursting out of the macrophage or neutrophil^4,5^. Therefore, understanding the cellular mechanisms that drive morphogenesis is crucial in defining its pathogenic traits.

In our previous studies^6–8^, we have demonstrated that during the yeast-to-hyphal transition the central carbon metabolism of *C. albicans* is globally downregulated. In particular, the pathways involved in the metabolism of alanine, *β*-alanine, aspartate, cysteine, histidine, glutamate, methionine, nitrogen, and nicotinate/nicotinamide, as well as the biosynthesis of acetyl-CoA are repressed. By using an isotope labelling experiment to trace the catabolism of the quorum sensing molecule phenylethyl alcohol, that represses hyphae formation under hyphae-inducing conditions, we have also found strong evidence for the involvement of NADP^+^/NADPH and NAD^+^/NADH in phenylethyl alcohol-mediated morphogenesis. This indicates that the redox balance of the fungal cells could be intrinsically associated with morphogenesis. Therefore, we decided to knock out the genes encoding NAD^+^-dependent glutamate dehydrogenase (*GDH2*) and NADPH-dependent glutamate dehydrogenase (*GDH3*) in order to assess the role of the cell’s redox balance and nitrogen metabolism in *C. albicans* morphogenesis.

**Fig. 1:**
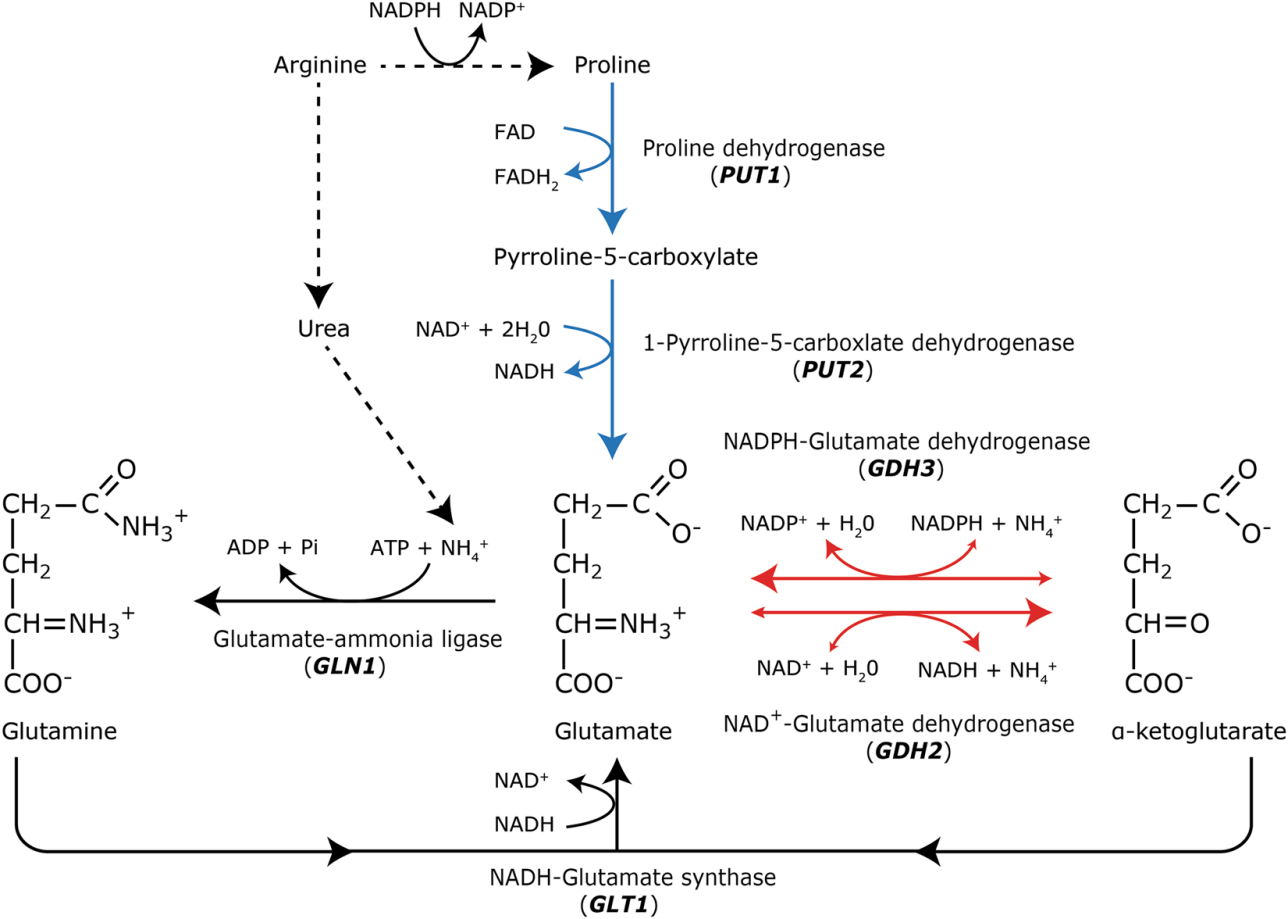
Three primary metabolic pathways for nitrogen assimilation and glutamate metabolism in *C. albicans*. Red lines: the reactions catalysed by glutamate dehydrogenases (*GDH2* and*GDH3*). Large arrows indicate reactions that are more favourable, thermodynamically. Black lines: the reactions catalysed by a combination of *GLN1* and *GLT1*, also referred to as GS and GOGAT in *S. cerevisiae*, respectively. Blue lines: the reactions catalysed by proline utilisation enzymes (*PUT1* and *PUT2*). Black dashed line: the reactions responsible for arginine degradation to proline and ammonia

*GDH2* and *GDH3* play central roles in nitrogen assimilation and excretion (Fig. 1), and in the maintenance of the redox balance of the cell^9–11^. Although the reactions catalysed by Gdh2 and Gdh3 are reversible, the Gdh2-catalysed reaction favours the deamination of glutamate—its catabolism to *α*-ketoglutarate and ammonium^12^. This reaction also generates NADH^13^. It is considered to be the final step in eukaryotic nitrogen excretion and very important in recycling oxidised NAD^+^. On the other hand, Gdh3 is predominantly involved in an anabolic reaction whereby inorganic ammonium is combined with *α*-ketoglutarate to form glutamate and, in the process, oxidises NADPH. Glutamate subsequently serves as an important nitrogen carrier for the biosynthesis of other amino acids via transamination reactions. In addition, dimorphic fungi such as *Benjaminiella poitrasii*, *Mucor racemosus*, and *Schizophyllum commune* have been reported to possess different NAD^+^- and NADPH-glutamate dehydrogenase activities when growing in the yeast and filamentous forms^14–16^. In addition, there are two alternative pathways involved in nitrogen assimilation in yeast cells (Fig. 1). The second pathway is mediated by the combined enzymatic activities of glutamate-ammonia ligase (often called glutamine synthase in *Saccharomyces cerevisiae*) and glutamate synthase, encoded by *GLN1* and *GLT1* in *C. albicans*, and known as *GS* and *GOGAT* in *S. cerevisiae*, respectively^17^. *GLN1* is predicted to catalyse the amination of glutamate to form glutamine. *GLT1* then transfers the amide group from glutamine to α-ketoglutarate, synthesising two molecules of glutamate^18^. The third pathway is proline utilisation which occurs in mitochondria where proline is oxidised to pyrroline-5-carboxylate by mitochondrial proline dehydrogenase (*PUT1*) which, in turn, is further oxidised to glutamate by 1-pyrroline-5-caboxylate dehydrogenase (*PUT2*)^19^. Although these two pathways have not been studied in *C. albicans* directly, they are likely to be present because orthologs of the genes encoding the *S. cerevisiae* enzymes have been found in the *C. albicans* genome (http://www.candidagenome.org//).

In this study, we used the *SAT1* flipper method^20^ to knock out the *GDH2* or *GDH3* gene in *C. albicans* followed by characterisation of growth under hyphae-inducing conditions. To further characterise the effect of the *GDH2* and *GDH3* gene deletions on *C. albicans* metabolism under hyphae inducing conditions, we combined metabolomics and isotope labelling with targeted quantification of nucleotides (e.g. ATP, NAD^+^, NADH, NADP^+^, and NAPDH). Identifying metabolic changes specific to the deletion of *GDH2* and *GDH3* genes will assist in the characterisation of the role of glutamate dehydrogenase in *C. albicans* morphogenesis, and hence its contribution to the virulence of this opportunistic pathogen.

## Results

### PCR verification of *GDH2*/*GDH3* genes knockout mutagenesis

The *SAT1* flipper disruption cassettes for *GDH2* and *GDH3* gene deletions were generated successfully by PCR. Portions upstream (443 bp) and downstream (338 bp) of the *GDH2* gene were amplified and fused with the 5269 bp *SAT1* fragment to form a disruption cassette (~6 kb) for *GDH2* gene deletion (Supplementary Fig. 2). Likewise, 527 bp of the upstream and 446 bp of the downstream regions of the *GDH3* gene were amplified and fused with the *SAT1* fragment to form a disruption cassette (~6 kb) for *GDH3* gene deletion (Supplementary Fig. 2).

To confirm that the *SAT1* flipper disruption cassettes had correctly replaced either the *GDH2* or *GDH3* gene after double crossover integrative transformation, the 5′ and 3′ junctions of disrupted alleles were PCR amplified. The successful allele disruptions were validated by PCR using primers C1, C2, C3, and C4 (Supplementary Fig. 1E) which generated the correct band sizes as illustrated in Supplementary Fig. 3. The PCR amplicon sizes for the 5′ check and 3′ check of *GDH2* gene disruption, 490 and 980 bp, matched the predicted values of 466 and 950 bp, respectively. Furthermore, the PCR amplicon sizes for the 5′ check and 3′ check of *GDH3* gene disruption, 600 and 790 bp, matched the predicted values of 607 and 751 bp, respectively.

To verify that the *SAT1* disruption cassettes were successfully excised from *GDH2/gdh2* and *GDH3/gdh3* strains, genomic PCR was used with primers C1 and C4 to amplify the region between the upper end of 5′ flank sequences to the lower end of 3′ flank sequences to make sure that both alleles of each gene were removed (Supplementary Fig. 4). The expected PCR amplicon sizes after the excision of disruption cassettes from *GDH2* and *GDH3* gene deletions were 1147 and 1191 bp, respectively. Both PCR amplifications of these regions yielded bands of ~1200 bp. These PCR reactions verified the excision of the *SAT1* disruption cassette. Thus, we confirmed correct construction of the *GDH2* and *GDH3* knockout strains.

### The morphologies of *gdh2*/*gdh2* and *gdh3*/*gdh3* mutant strains, and the parental strain, grown on different media

**Fig. 2:**
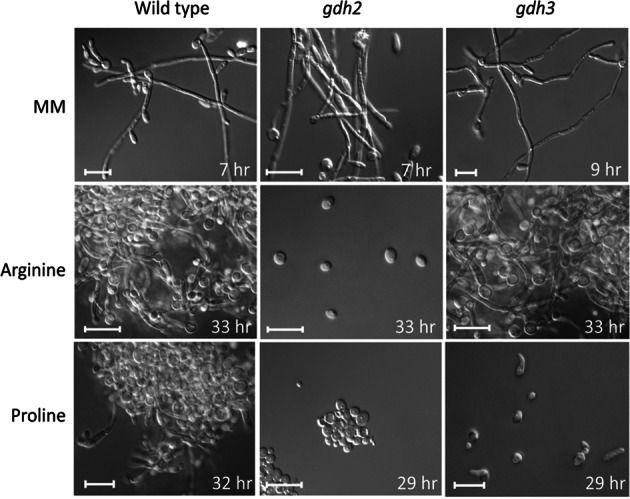
The morphologies of the *gdh2/gdh2*, *gdh3/gdh3*, and wild-type strains of *C. albicans* grown on various media. Cells were incubated at 37 °C and examined at the end of exponential growth phase. MM medium is minimum mineral medium with 1% glucose. Arginine or proline media are minimum mineral medium without any carbon and nitrogen source but supplemented with either arginine or proline. The wild-type strain is *C. albicans* SC5314. The images were obtained by Nomarksi contrast microscopy with ⨯100 magnification. The scale bars on each panel represent 10 μm

There was no obvious difference in hyphal development between the wild-type (parental) and the mutant *C. albicans* strains when cultured in minimum mineral (MM) medium (Fig. 2), or under other hyphae-inducing conditions (Supplementary Fig. 7). However, when proline or arginine was provided as sole carbon and nitrogen sources, distinct differences were seen. In proline medium, the *gdh3*/*gdh3* mutant strain failed to form filaments, whilst the wild-type developed hyphae (Fig. 2). In contrast, in arginine medium both the *gdh3*/*gdh3* and wild-type strains completely shifted from yeast to hyphal growth. Interestingly, both the *gdh3*/*gdh3* mutant and the wild-type strains showed abundant chlamydospores when incubated in arginine medium (Fig. 2). These structures are related to stress and starvation in *C. albicans*. These experiments demonstrated that the *gdh3*/*gdh3* mutant was locked in the yeast morphology when proline was supplied as the sole carbon and nitrogen source.

### The growth kinetics of *gdh2*/*gdh2* and *gdh3*/*gdh3* mutant strains and the parental *C. albicans* strain

**Fig. 3:**
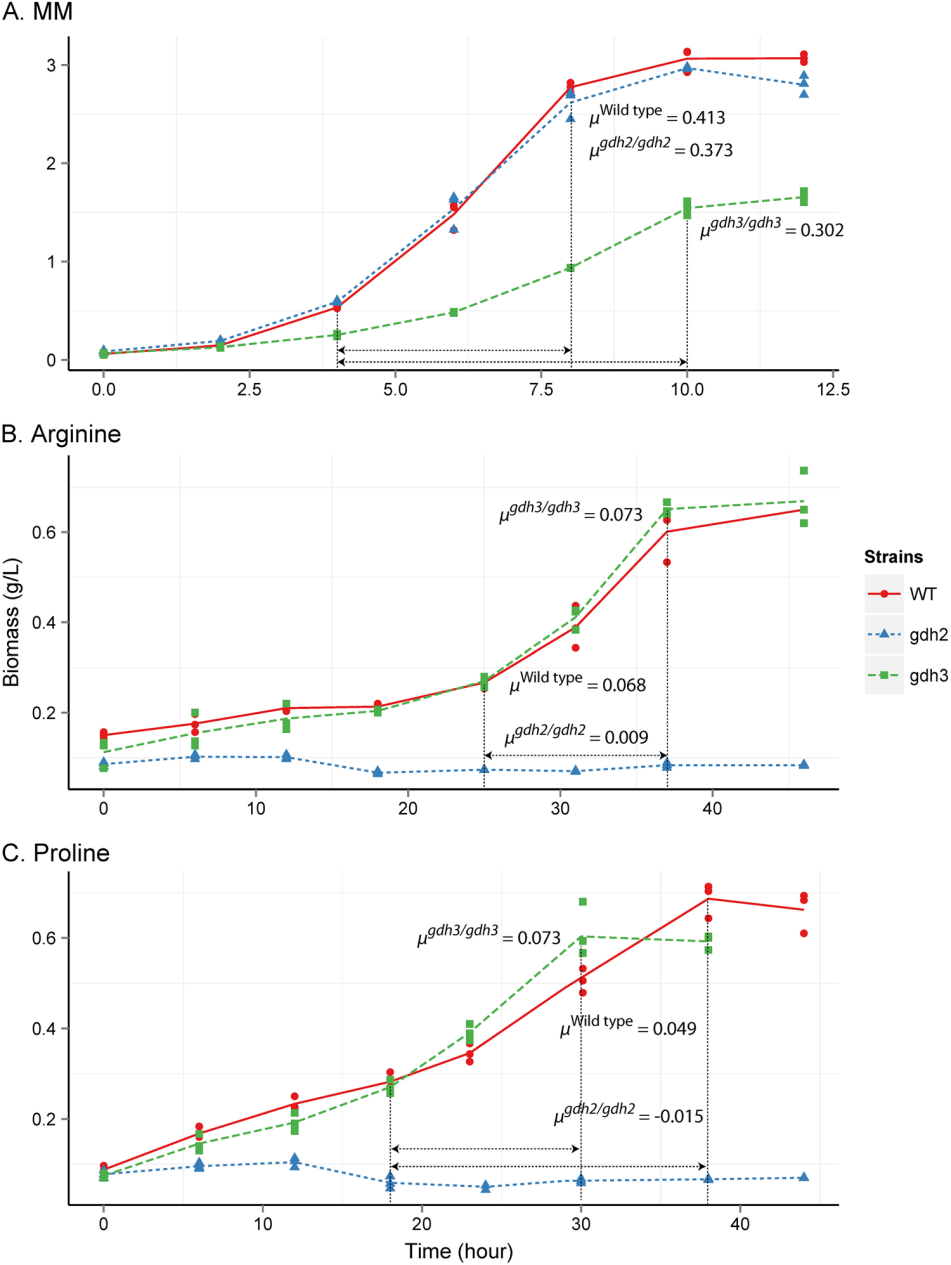
Growth curves and growth rates for *gdh2*/*gdh2*, *gdh3*/*gdh3*, and wild-type strains of *C. albicans* grown on various media. **a** minimum mineral medium, **b** arginine medium, and **c** proline medium. All cultures were incubated at 37 °C with shaking (200 rpm) until the stationary phase was reached. *µ* represents the exponential growth rate (h^−1^). Five experimental replicates were collected from each growth medium

To characterise the effect of *GDH2* and *GDH3* gene deletions on the growth kinetics of *C. albicans*, we monitored the growth of the wild-type and mutant strains on MM, arginine, and proline media at 37 °C under continuous agitation (200 rpm) (Fig. 3). When the fungal cells were grown on MM medium, there was no significant difference in growth rate (~0.40 h^−1^) between the wild-type and *gdh2/gdh2* strains. The *gdh3*/*gdh3* mutant, however, showed a 24% reduction in its growth rate (0.302 h^−1^) compared to wild-type cells.

On the other hand, the *gdh3*/*gdh3* mutant showed similar growth kinetics to the wild-type cells when grown on arginine as the sole carbon and nitrogen source, but the *gdh2*/*gdh2* strain showed minimal growth (0.009 h^−1^) and biomass yield (0.016 g L^−1^) compared to the wild-type strain (growth rate = 0.068 h^−1^, biomass = 0.50 g L^−1^). Furthermore, the *gdh2*/*gdh2* mutant failed to grow on proline as the sole carbon source. This indicated that the *gdh2*/*gdh2* mutation significantly impaired *C. albicans* utilisation of arginine or proline as the sole carbon and nitrogen source (Fig. 3). The relatively slower growth rate and lower growth yield of the wild-type and *ghd3*/*gdh3* strains on proline or arginine medium, compared to MM medium, can be explained by the fact that amino acids are poor carbon and nitrogen sources in comparison to the glucose and ammonia present in MM medium.

### The extracellular metabolite profiles of *gdh2*/*gdh2* and *gdh3*/*gdh3* mutant strains and the parental *C. albicans* strain

Thirty-seven metabolites were identified in the spent media of *C. albicans* culture samples (Table 1), and the concentrations of 24 and 19 of these metabolites were significantly different (*p* < 0.05) between wild-type and mutant strains when grown on proline and MM media respectively (Supplementary Fig. 5). In contrast, only 3 extracellular metabolites, 2-isopropylmalate, 4-aminobenzoate, and glutamate, showed significant differences between wild-type and *gdh3*/*gdh3* strains cultured on arginine medium. Interestingly, when growing in proline medium, in which the *gdh3/gdh3* mutant cells failed to undergo morphogenesis, they exhibited an overall reduction in extracellular metabolite levels when compared to the wild-type, with the exception of nicotinate, malate, and succinate levels.

**Table 1:** Intracellular and extracellular metabolites associated with the growth of *gdh2/gdh2*, *gdh3/gdh3*, and wild-type strains of *C. albicans* in different media

Classification of metabolites	Intra^a^	Extra^b^	Metabolites
Amino acids	17	6	Alanine*, asparagine, aspartate, cysteine, glutamate*, glutamine, glycine, histidine, isoleucine*, leucine*, lysine, phenylalanine, proline*, serine, tryptophan, tyrosine and, valine*
Amino acid derivatives	10	2	Creatinine, cystathionine, l-2-aminoadipiate, *N*-acetylglutamate*, norvaline, ornithine*, *O*-acetyl-l-serine, *S*-adenosyl-l-homocysteine, and pyroglutamate, β-alanine
TCA cycle intermediates	6	4	Citrate*, succinate*, *cis*-aconitate, isocitrate, malate*, and 2-oxoglutarate*
Fatty acids	17	4	*cis*-11-Eicosadienoate, *cis*-11,14-eicosadienoate, *cis*-11,14,17-eicosatrienoate, decanoate, hexanoate, linoleate, laurat, margarate, myristate, oleate*, palmitate*, palmitoleate*, pentadecanoate, isopalmitate, stearate*, 3-hydroxyoctanoate, linolenate, and γ-linolenate
Glycolytic intermediates	2	0	Pyruvate and phosphoenolpyruvate
Cofactors and vitamins	3	1	NADP/NADPH, nicotinate*, and 4-amino-n-butyrate
Antioxidants	1	0	Glutathione
Others	12	10	Citraconate*, citramalate*, glutarate, itaconate*, lactate*, malonate*, quinate*, 5-oxotetrahydrofuran-2-carboxylate*, 4-aminobenzoate*, oxalate*, putrescine, and p-toluate
Metabolites only found in extracellular media	0	10	Arachidate*, behenate*, *cis*-vaccenate*, EDTA*, glyoxylate/glyoxalate*, tridecane*, 2-oxoadipate*, 2-isopropylmalate*, 3,5-diiodo-l-tyrosine* and 4-hydroxyphenylacetate*
Total number of identified metabolites	68	37	

Asterisks indicate metabolites only found in extracellular media

^a^Intra: number of intracellular metabolites identified in any samples

^b^Extra: number of extracellular metabolites identified in any samples

### The intracellular metabolite profiles of *gdh2*/*gdh2*, *gdh3*/*gdh3*, and parental strains of *C. albicans*

**Fig. 4:**
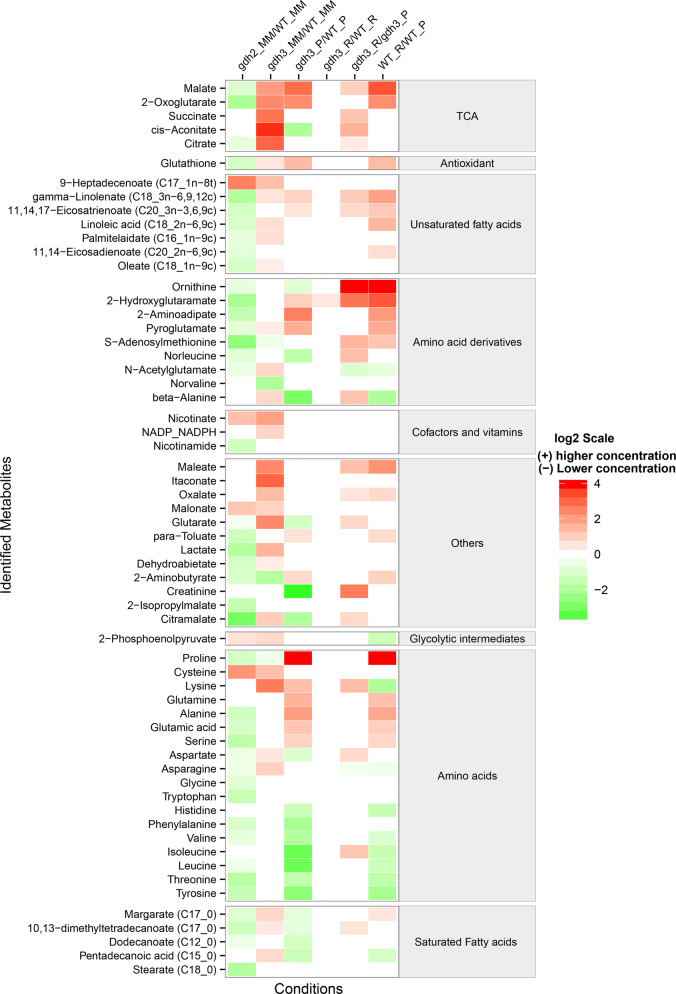
The ratio of intracellular metabolite concentrations between *C. albicans* strains when cultured on arginine (R), proline (P), or minimum mineral (MM) media. The metabolite concentrations relative to those in the wild-type (except column 5 which is relative to *gdh3* cells grown in proline medium) have been plotted using a log_2_ scale. The red colour (positive values) indicates that metabolite concentrations were higher in numerator strains than the denominator (comparator) strains, while green shades (negative values) indicate reduced concentrations in numerator strains compared to the denominator strains. Only the metabolites for which there was a statistically significant change in concentration between the wild-type and mutant strains (Tukey’s honest significance test, *p*-value < 0.05) are shown

In order to understand how *GDH2* and *GDH3* gene knockouts influence the metabolic changes associated with *C. albicans* morphogenesis, we compared the intracellular metabolite profiles between wild-type and mutant strains cultured in MM, arginine, or proline media. There were over 100 metabolites detected in the intracellular extracts and 68 of them were identified by our in-house mass spectral library (Table 1). Among these, the relative concentrations of 62 metabolites were significantly influenced by either *GDH2* or *GDH3* gene deletions (Fig. 4). Interestingly, when the *gdh2*/*gdh2* mutant utilised glucose as the sole carbon source (MM medium), most of the intracellular metabolites were found at lower concentrations than in the wild-type strain except for cysteine, malonate, nicotinate, 9-heptadecenoate, and 2-phosphoenolpyruvate. In contrast, the *gdh3*/*gdh3* mutant showed overall higher concentrations of intracellular metabolites than the wild-type strain when grown in MM medium, except for 2-aminobutyrate and norvaline.When grown in a proline medium, the *gdh3*/*gdh3* mutant revealed a significantly higher concentration of glutamate family metabolites (e.g., glutamate, glutamine, and proline) than the wild-type, whilst asparagine, histidine, phenylalanine, valine, leucine, tyrosine, and aspartate family metabolites (e.g., aspartate, isoleucine, and threonine), as well as all saturated fatty acids detected, were at lower concentrations in the *gdh3*/*gdh3* mutant. Coincidentally, valine, leucine, tyrosine, phenylalanine, aspartate, and isoleucine are synthesised by a transamination reaction in which these amino acids gain their amino group by converting glutamate to α-ketoglutarate. When grown in an arginine medium, on the other hand, there were only a few intracellular metabolites for which their concentrations were significantly changed comparing the *gdh3*/*gdh3* and wild-type cells, suggesting that the *gdh3*/*gdh3* mutant cells were in a similar metabolic state to the wild-type cells under this environmental condition (Fig. 4). Furthermore, a comparison of the metabolite profiles for *gdh3*/*gdh3* mutants grown in arginine and proline media (Fig. 4, column 5) demonstrated that 20 intracellular metabolites were at higher concentrations in the *gdh3*/*gdh3* mutant cultured in the arginine medium. These metabolites included a range of intermediates from the central carbon metabolism including tricarboxylic acid (TCA) cycle intermediates, amino acids, saturated fatty acids, and unsaturated fatty acids. There were only two metabolites, asparagine and *N*-acetylgluatmate, detected at a lower concentration in the *gdh3*/*gdh3* cells grown on arginine medium than on proline medium. We investigated how arginine and proline media affected the intracellular metabolites of *gdh3*/*gdh3* and wild-type strains differently (Fig. 4, columns 5 and 6). We detected a lower arginine/proline media ratio (lower metabolite concentrations in arginine medium compared to proline medium) for 2-oxoglutarate, linoleate, 11,14-eicosadienote, proline, 2-aminoadipate, pyroglutamate, para-toluate, 2-aminobutyrate, lysine, glutamine, alanine, glutamate, serine, and margarate in *gdh3*/*gdh3* mutants compared to wild-type. In contrast, we observed a higher arginine/proline media ratio (higher metabolite concentrations in arginine medium compared to proline medium) for succinate, *cis*-aconitate, citrate, norleucine, β-alanine, creatinine, citramalate, lysine, aspartate, isoleucine, and 10,13-dimethyltetradecanoate in *gdh3*/*gdh3* mutants compared to wild type (Fig. 4, columns 5 and 6). Changes in these metabolite ratios are likely to be the result of the yeast to hyphae morphological transition.

### Effect of *GDH2* and *GDH3* gene deletions on the metabolic state of *C. albicans* under hyphae-inducing conditions

**Fig. 5:**
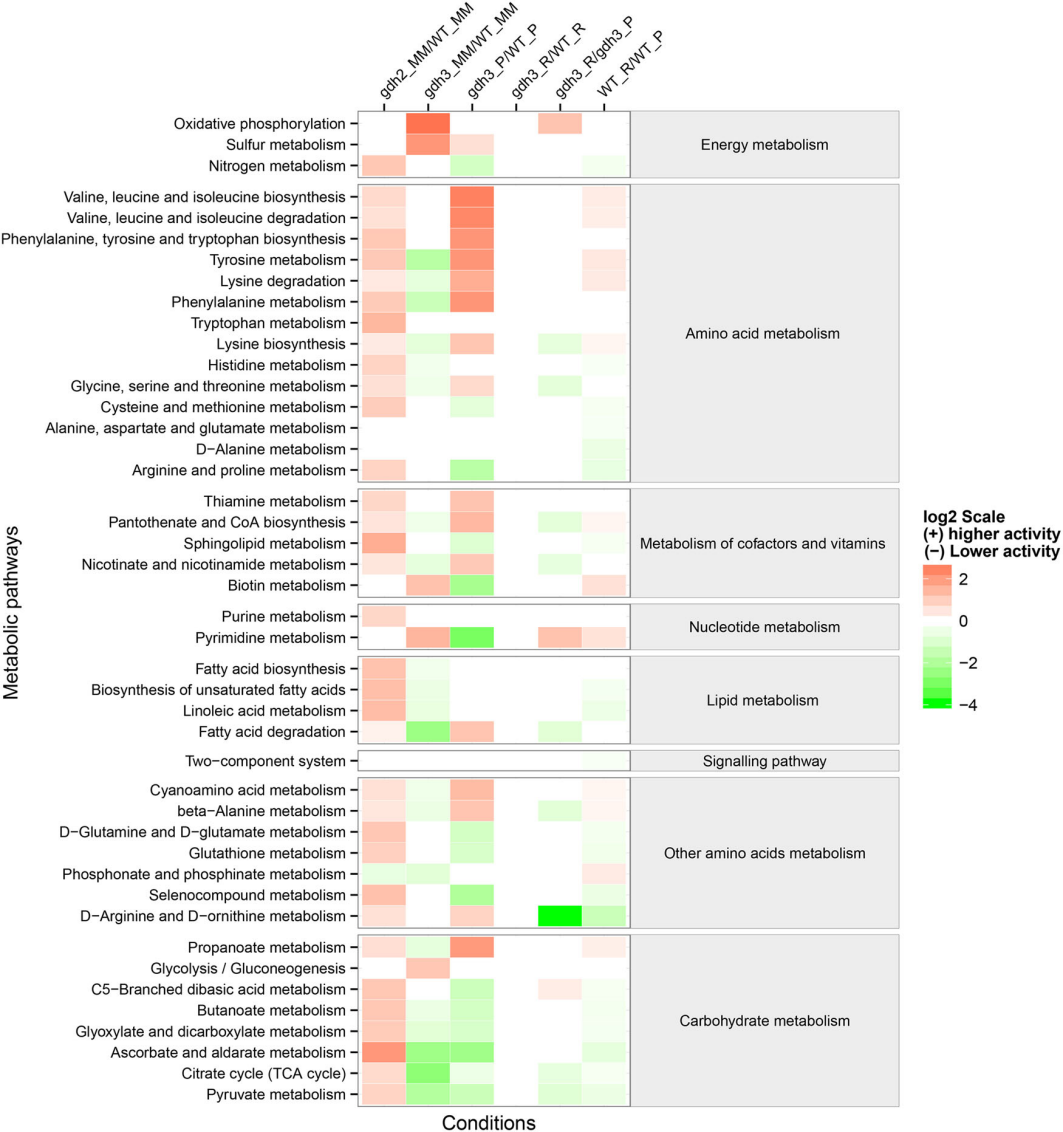
Activities of *C. albicans* metabolic pathways predicted from intracellular metabolomics data from the wild-type (WT) and *gdh2*/*gdh2* or *gdh3*/*gdh3* strains grown in various culture media. MM is minimum mineral medium, P is proline medium, and R is arginine medium. The metabolic pathway activities relative to the denominator strains are shown using a log_2_ scale. Red colours (positive values) mean pathways that were upregulated in numerator strain compared to the denominator (comparator) strain. Green colours (negative values) indicate pathways that had their activity downregulated in numerator strain compared to the denominator strain. Only the metabolic pathways for which there was a statistically significant change in activity (Tukey’s honest significance test, *p*-value < 0.05) are shown

The quantification of intracellular metabolites was used to generate a comparative metabolic activity profile for the wild-type and mutant cells grown in MM, arginine, and proline media. Out of the 36 metabolic pathways that showed significant changes in metabolic activity in response to the *GDH2* gene deletion when cells were cultured in MM medium, all except phosphonate and phosphinate metabolism were upregulated (Fig. 5). On the other hand, 22 metabolic pathways were significantly downregulated in *GDH3*-deleted cells cultured in MM medium and only oxidative phosphorylation, sulphur metabolism, biotin metabolism, pyrimidine metabolism, and glycolysis/gluconeogenesis were upregulated. This suggests that the *GDH2* and *GDH3* genes may play opposite metabolic roles in *C. albicans*. Interestingly, no metabolic pathways appeared to show significantly different activity between the *gdh3*/*gdh3* mutant and the wild-type strain when grown in arginine medium (Fig. 5, column 4). By comparing the metabolic pathway activities for the *gdh3*/*gdh3* mutant grown on arginine medium as opposed to on proline medium with the wild-type strain under those growth conditions, there was a greater global metabolic reprogramming for the wild-type cells when grown on arginine compared to growth on proline (Fig. 5, columns 5 and 6). Although the wild-type cells retained the same filamentous form in both culture media, by comparing the metabolic activities between arginine and proline media, 16 metabolic pathways were downregulated, whilst 11 metabolic pathways were upregulated for the wild type compared to the *gdh3*/*gdh3* mutant.

### ^13^C-label distribution through the metabolite profile of *C. albicans* cells grown on 30% U-^13^C_6_ arginine or 30% U-^13^C_5_ proline as both the sole carbon and nitrogen source, respectively

**Fig. 6:**
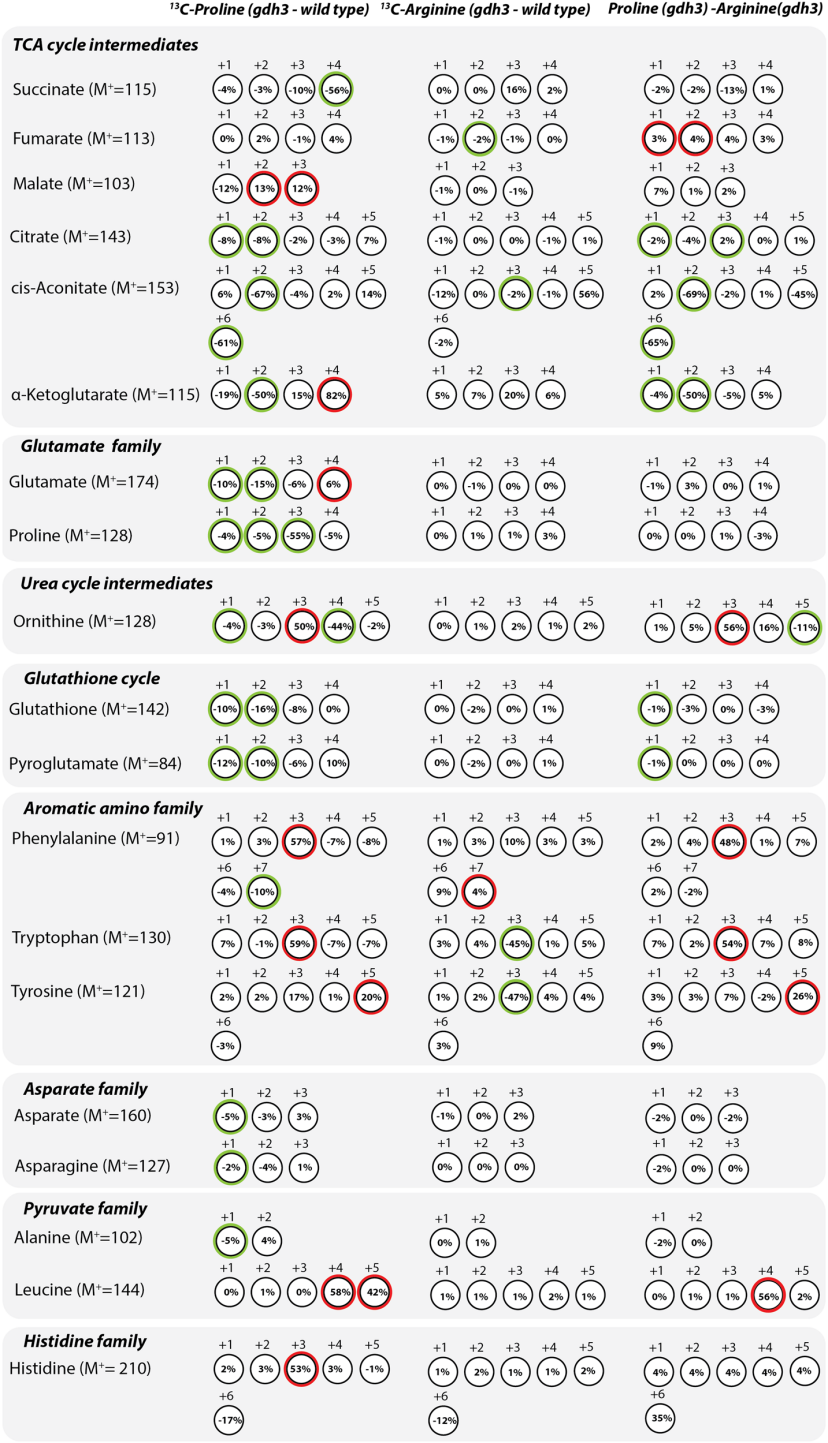
The incorporation of ^13^C carbon atoms from U-^13^C_5_ proline or U-^13^C_6_ arginine into metabolites from the wild-type and mutant strains of *C. albicans*. The figure indicates the percentage difference of ^13^C-labelling between two cell types when utilising 30% U-^13^C_6_ arginine or 30% U-^13^C_5_ proline as both the sole carbon and nitrogen source, respectively. M^+^ is the main molecular ion of an identified metabolite. +1 is 1 *m*/*z* higher than the M^+^. Only the metabolite ions for which there was a statistically significant change in the percentage of ^13^C-atom between the cell types (*p*-value < 0.05 and >2-fold differences) are highlighted with coloured circles. Green circles represent lower isotope labelling in the first strain. Red circles indicate higher isotope labelling in the first strain

In order to track how mutants catabolise arginine and proline under hyphae-inducing conditions, we supplied mutant and wild-type strains with 30% ^13^C-labelled arginine or 30% ^13^C-labelled proline as the sole carbon source. Our premise was that increased labelling of a metabolite indicates a higher rate of biochemical conversion from the labelled carbon source to the metabolite. Conversely, a decrease in labelling indicates reduced metabolic flux. We found that many metabolites were significantly reduced in one and/or two ^13^C-labelling (see Fig. 6: M + 1 and M + 2, which mean one carbon and two carbons labelled, respectively) in the *gdh3*/*gdh3* mutant growing in ^13^C-labelled proline medium, relative to wild-type cells (Fig. 6, first column). These included TCA cycle intermediates such as citrate, *cis*-aconitate, *α*-ketoglutarate, and various amino acids including glutamate, proline, aspartate, asparagine, and alanine as well as glutathione, pyroglutamate, and ornithine. Moreover, we observed an increased C^13^-labelling of TCA cycle intermediate malate (Fig. 6). Interestingly, some metabolites such as malate, α-ketoglutarate, ornithine, glutamate, ornithine, phenylalanine, tryptophan, tyrosine, leucine, and histidine had higher percentage ^13^C-labelling in three or more carbon atoms (M+ ≥ 3) in the *gdh3*/*gdh3* mutant compared to wild-type cells grown in 30% U-^13^C_5_ proline medium. In contrast, when utilising 30% U-^13^CR_6_-labelled arginine as the sole carbon source, there were only a few differences between the metabolic labelling in *gdh3*/*gdh3* and wild-type cells that included two TCA cycle intermediates (fumarate and *cis*-aconitate) and all three aromatic amino acids (phenylalanine, tryptophan, and tyrosine). The difference in aromatic amino acid metabolism may affect the level of quorum sensing molecules, because both phenylalanine, tryptophan, and tyrosine are precursors for the biosynthesis of phenylethyl alcohol, typtophol, and tyrosol respectively. Lastly, in general there were similar labelling patterns when *gdh3*/*gdh3* mutants were compared with the wild-type strain grown on proline as when *gdh3*/*gdh3* cells grown in proline and in arginine media were compared (column 1 and column 3 in Fig. 6).

### Cofactor concentrations in *C. albicans* wild-type and mutant strains

**Fig. 7:**
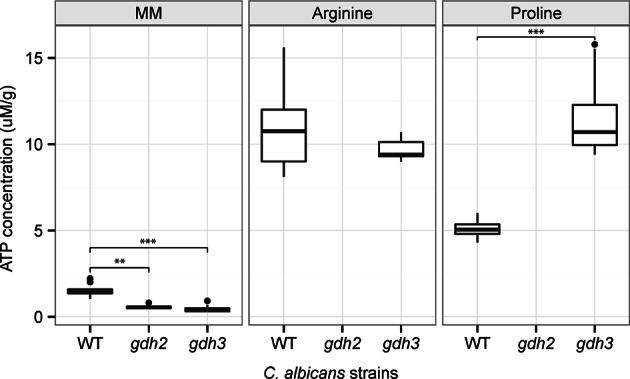
ATP concentrations in the wild-type (WT)*, gdh2*/*gdh2*, and *gdh3*/*gdh3* strains of *C. albicans* incubated in the three different media. The ATP pools in mutant cells grown in (MM) minimum mineral and proline media were significantly different from the wild-type but not cells cultured in arginine medium. ATP was not measured in the *gdh2*/*gdh2* mutant incubated in arginine or proline media because this mutant was unable to grow under these conditions. The unit for ATP concentration is μmole per gram of cell biomass. Fifteen experimental replicates were performed for each group. The distributions of boxplot are minimum, 25th percentile, median, 75th percentile, and maximum (from bottom to upper direction). Black dots are outliers (>1.5 times of interquartile range). **Tukey’s honest significance test (*p*-value < 0.05) and ***(*p*-value <0.001)

**Fig. 8:**
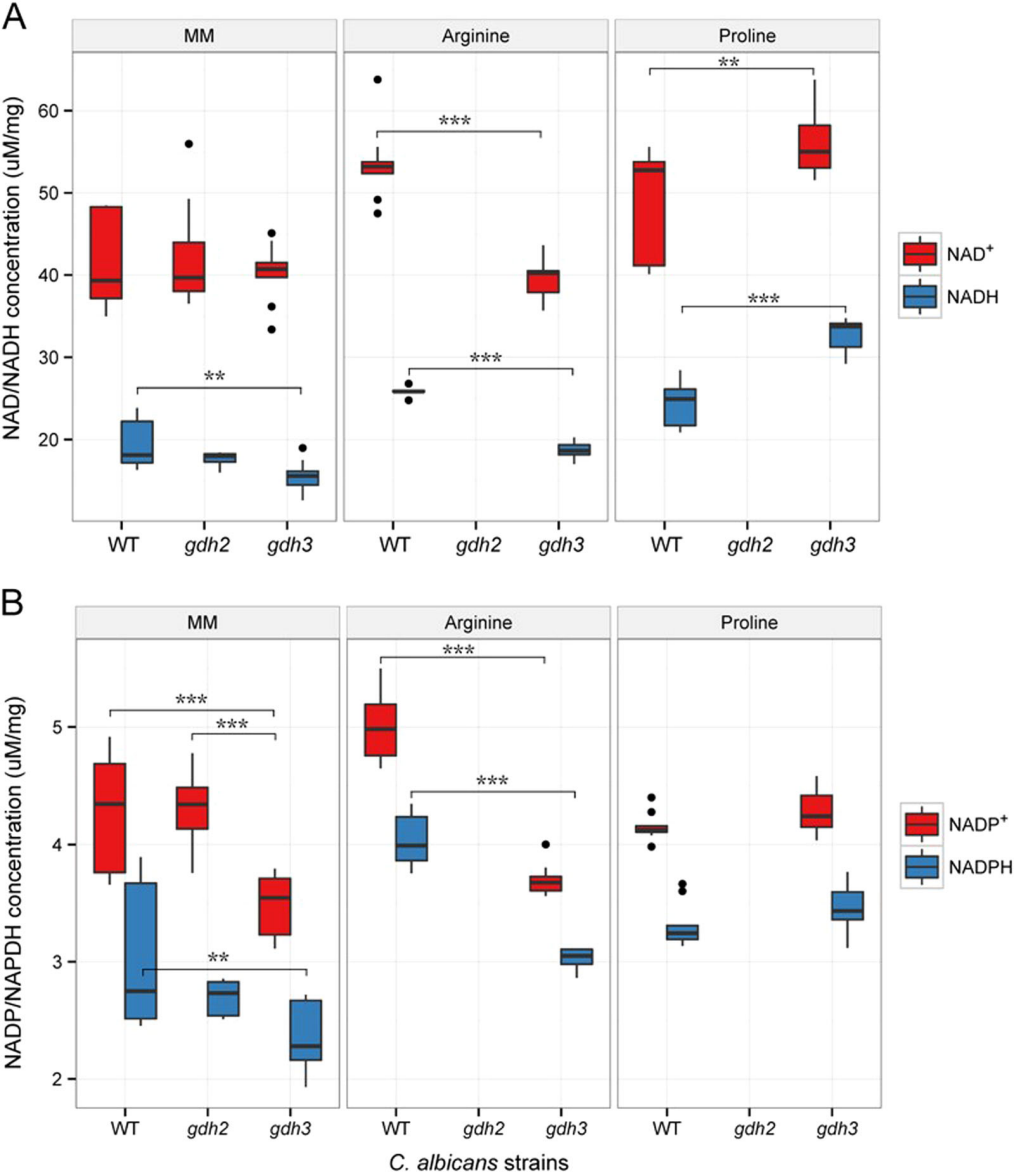
Cofactor concentrations in the wild-type (WT), *gdh2*/*gdh2*, and *gdh3*/*gdh3* strains of *C. albicans* incubated in arginine, proline, or minimum mineral (MM) media. **a** The concentration of NAD^+^ (red boxes) and NADH (blue boxes) found in *C. albicans* mutant and wild-type strains. **b** The concentration of NADP^+^ (red boxes) and NADPH (blue boxes) detected in *C. albicans* mutant and wild-type strains. Cofactor concentrations were not measured in the *gdh2*/*gdh2* mutant under arginine and proline media because this mutant was unable to grow under these conditions. The unit for NADP/NADPH concentration is μmole per gram of cell biomass. Nine experimental replicates were collected and measured from each group. The distributions of boxplot are minimum, 25th percentile, median, 75th percentile, and maximum (from bottom to upper direction). Black dots are outliers (>1.5 times of interquartile range). Tukey’s honest significance test **(*p*-value *<* 0.05) and ***(*p*-value < 0.001)

The concentrations of ATP, NAD^+^, NADH, NADP^+^, and NADPH in *C. albicans* cells were measured in order to determine the energetic and redox state of the different strains under different growth conditions (Figs 7 and 8). The oxidised forms of NAD^+^ and NADP^+^ were found at higher concentrations than the reduced forms in samples from all *C. albicans* strains under the different growth conditions (Fig. 8). The *gdh3*/*gdh3* mutant incubated in arginine medium exhibited significantly lower NAD^+^, NADH, NADP^+^, and NADPH concentrations (*p* < 0.001) compared to the wild-type cells (Fig. 8), while ATP remained at a similar concentration (Fig. 7). In contrast, the *gdh3*/*gdh3* mutant showed a higher concentration of ATP, NAD^+^, and NADH, but not NADP^+^ or NADPH, when incubated in proline medium when compared to the wild-type strain (Figs 7 and 8). The higher concentrations of ATP, NAD^+^, and NADH could explain why the *gdh3*/*gdh3* mutant remained in the yeast form in arginine medium (hyphae-inducing conditions). In MM medium, only the *gdh3*/*gdh3* mutant showed a significantly lower concentration of NADH, NADP^+^, and NADPH than the wild-type strain. Furthermore, when the ratios of NAD^+^ to NADH in wild-type and mutant strains were compared we detected a significantly lower ratio of NAD^+^ to NADH in the *gdh3/gdh3* mutant than in the wild-type strain when incubated in proline medium, while a higher ratio of NAD^+^ to NADH in the *gdh3/gdh3* mutant than in the wild-type strain in MM medium (Supplementary Fig. 6). The decreased ratio of NAD^+^ to NADH in the *gdh3*/*gdh3* mutant indicates that cofactor equilibrium may be important for the morphogenesis of *C. albicans*.

## Discussion

This study implicates *GDH2* and *GDH3* gene knockout mutagenesis, metabolomics, and isotope labelling approaches to elucidate how the fungus *C. albicans* changes its cellular metabolism during the yeast-to-hyphal transition. There were three major comparisons performed in this study in order to determine the role of *GDH2* and *GDH3* in *C. albicans* growth and morphogenesis. Firstly all three strains, the *GDH2* and *GDH3* deletion mutants and the wild-type strain, grown in MM medium were compared to investigate the metabolic differences between mutant and wild-type strains during the morphological transition (*gdh2*-MM vs WT-MM, *gdh3*-MM vs WT-MM). Secondly we compared the metabolic profiles of the *gdh3*/*gdh3* mutant and wild type when grown in arginine and proline media (*gdh3*-P vs WT-P, *gdh3*-R vs WT-R). Since the *gdh2*/*gdh2* mutant was unable to grow, when arginine and proline were provided as the sole carbon and nitrogen sources, this strain could not be used in this experiment. The last comparison was of the concentrations of different metabolites between arginine and proline media for *gdh3*/*gdh3* mutant compared to those in the wild-type strain under the same conditions (comparison between *gdh3*-R/*gdh3*-P and WT-R/WT-P). The purpose of the last comparison was to determine the metabolic differences in the *gdh3*/*gdh3* mutant caused by different growth morphologies. We could do this because the *gdh3*/*gdh3* mutant was unable to undergo morphogenesis in the proline medium but became highly filamentous in the arginine medium (Fig. 2). This enabled us to remove the metabolic effect from the use of different media.

We expected that the *gdh2*/*gdh2* and *gdh3*/*gdh3* mutations would induce a significant alteration in the redox potential of the *C. albicans* cells when we deleted these genes, as previously observed in *S. cerevisiae*^9^, and hypothesised that this would also affect morphogenesis. Indeed, we found that the concentrations of oxidised and reduced forms of NAD and NAPD were significantly changed in the *gdh2*/*gdh2* and *ghd3*/*gdh3* mutants compared to the wild-type strain in MM, arginine and proline media. The deletion of the *GDH2* or *GDH3* gene in *C. albicans*, however, did not seem to affect its morphogenetic ability greatly in MM medium. This implied that the redox potential of the cell alone may not be enough to affect the morphogenesis of *C. albicans*. On the other hand, the yeast form of the *gdh3*/*gdh3* mutant showed a significantly higher intracellular concentration of ATP, NAD^+^, NADH, NADP^+^, and NADPH compared to the filamentous form of the wild-type strain in proline medium. These results supported the hypothesis we previously proposed^8^ that once the metabolic activity of yeast cells is temporarily disrupted by environmental stresses, the central carbon metabolism of *C. albicans* is globally downregulated. It is proposed that this downregulation of the central carbon metabolism triggers changes to the growth form as a result of less energy being available, and consequently impacts filamentous growth. We speculated that hyphae exhibit lower metabolic activities due to the necessity of utilising alternative carbon sources in contrast to the dominance of sugar metabolism in the yeast form. *C. albicans* often lives in the gastrointestinal tract of its mammalian host and on epithelial surfaces, where there are diverse and can be abundant carbon sources. Whilst competition with other microorganisms for nutrients is intense, an ability to assimilate alternative carbon and nitrogen sources, and to enable directional growth with filament development, is no doubt an advantageous survival strategy. Indeed, the *gdh3*/*gdh3* mutant showed reduced assimilation of proline as a carbon and nitrogen source, and with increased ATP concentrations, this may be the reason that the *gdh3*/*gdh3* mutant stayed in the yeast form.

Although the *gdh3*/*gdh3* mutant demonstrated similar growth behaviour as the wild-type strain in proline and arginine media, it showed attenuated growth in MM medium. We suggest that this is because the *gdh3*/*gdh3* mutant is unable to efficiently assimilate nitrogen sources into the central carbon metabolism. Gdh3 favours the assimilation of ammonium through the anabolic reaction with *α*-ketoglutarate to form glutamate. Since ammonium is the only nitrogen source in MM medium, the *gdh3*/*gdh3* mutant may rely on Gdh2 to incorporate ammonium into glutamate. This process will consume NADH and compromise the use of NADH for the production of ATP via the electron transport chain. Indeed, we observed a lower ratio of NADH to NAD^+^ (Supplementary Fig. 6A) and a lower level of ATP (Fig. 7) in the *gdh3*/*gdh3* mutant when it was grown in MM medium. The metabolic pathway analysis (Fig. 5) also predicted a global downregulation of central carbon metabolism in the *gdh3*/*gdh3* mutant compared to the wild-type cells in MM medium. On the other hand, the *ghd3*/*gdh3* mutation did not reduce growth rate in either the arginine or proline medium compared to the wildtype (Fig. 3). We propose this is because Gdh2 is still present and has a high affinity for the enzymatic deamination of glutamate into α-ketoglutarate. Therefore, it is not surprising that the *GDH3* deletion did not influence growth significantly because it is substituted by Gdh2.

The *gdh2*/*gdh2* mutant failed to utilise arginine and proline as both the sole carbon and nitrogen sources. Indeed, the biomass of the *gdh2*/*gdh2* mutant gradually reduced over 40 h of incubation in the proline and arginine media (Fig. 3). Both arginine and proline belong to the glutamate family of amino acids. When these amino acids are used as carbon and nitrogen sources, they must be converted into glutamate, followed by a transamination reaction into α-ketoglutarate and ammonia, which is then catabolised by glutamate dehydrogenase. Therefore, the deletion of the *GDH2* gene will certainly impact on the catabolism of arginine and proline, considering that Gdh2 is the main enzyme capable of converting glutamate into α-ketoglutarate through the reduction of NAD^+^. Furthermore, the inability to utilise arginine and proline could also suggest that both *PUT* and *GLN1/GLT1* pathways (Fig. 1) are dysregulated in response to the *GDH2* gene deletion, or these pathways are unable to generate sufficient glutamate inside the cells and the large pool of free NADP^+^ required to overcome the thermodynamic barrier inherent in using the still present Gdh3, which is usually an anabolic enzyme with a higher affinity for α-ketoglutarate than for glutamate. The lack of central carbon and nitrogen catabolism not only leads to energy deficiency but also reduces the supply of the main metabolite anabolic precursors such as pyruvate, acetyl-CoA, glucose-6-phosphate, and erythrose 4-phosphate used for biomass biosynthesis^21^. These effects are clearly demonstrated by growth attenuation (Fig. 3) and the lack of hyphal formation (Fig. 2) in the *gdh2*/*gdh2* mutant when proline or arginine were supplied as the sole carbon and nitrogen sources. In contrast, the *gdh2*/*gdh2* mutant can grow in MM medium because α-ketoglutarate is supplied from glucose via glycolysis.

We postulate that NAD^+^/NADH disequilibrium in the *gdh3*/*gdh3* mutant led to a different proline catabolism in the mutant compared to the wild-type strain. Our isotope labelling results (Fig. 6) demonstrated that the *gdh3*/*gdh3* mutant exhibited a lower percentage of ^13^C labelling of one to two carbon atoms in metabolites while a higher percentage of ^13^C labelling for three or more carbon atoms in metabolites for most of TCA cycle intermediates and amino acids when compared to the wildtype. This result could be explained by the fact that catabolism of proline in the *gdh3*/*gdh3* mutant involves only NAD^+^-dependent reactions, including the Gdh2- and Put2-catalysed reactions. The *PUT2* gene encodes pyrroline 5-carboxylate dehydrogenase which catalyses the conversion of pyrroline-5-carboxylate and NAD^+^ to glutamate (Fig. 1). This reaction is crucial for proline degradation when used as the sole source of carbon and nitrogen. Therefore, when proline is the only carbon and nitrogen source, the *gdh3*/*gdh3* cells would probably be experiencing a serious shortage of the NAD^+^ required to catabolise proline via the *PUT* pathway. We found an accumulation of proline, glutamate, 2-oxoglutarate, malate, and a lower NAD^+^ to NADH ratio inside the *gdh3*/*gdh3* mutant relative to wild-type cells when grown in proline medium. This indicated a lower influx of proline into the TCA cycle via the glutamate to α-ketoglutarate route. On the other hand, the wild-type strain could relieve the high NAD^+^ usage by employing Gdh3 which is dependent on NADP^+^. There are also alternative proline catabolic pathways that cleave proline into two-carbon molecules, yet are NAD^+^ dependent, so these metabolic pathways are suppressed in the *gdh3*/*gdh3* mutant. Since preferred nitrogen sources, such as ammonia and nitrogen were not present when proline was used as the sole carbon and nitrogen source, catabolite repression of the proline utilisation pathway (*PUT1* and *PUT2*) would not have occurred^19^. Surprisingly, a higher concentration of ATP was detected in the *gdh3*/*gdh3* mutant than in the wild-type strain. This could result from the higher NADH production by Gdh2 as well as an upregulation of β-oxidation. In proline medium, we observed a lower concentration of saturated fatty acids (Fig. 4) and higher metabolic activity involved in fatty acid degradation (Fig. 5) in the *gdh3*/*gdh3* mutant than in the wild-type strain. Since β-oxidation catabolises fatty acids into NADH, FADH_2_, and acetyl-CoA, which enters the TCA cycle, upregulated β-oxidation results in the degradation of fatty acids and increased ATP production.

Why is hyphal formation in the *gdh3*/*gdh3* mutant only suppressed in proline medium but not in arginine medium when both amino acids belong to the glutamate amino acid family? We also observed no significant differences in the metabolic pathway activity (Fig. 5) or cofactor ratio (Supplementary Fig. 6A) between the *gdh3*/*gdh3* mutant and wild-type strain when grown in arginine medium. The in silico model of the *C. albicans* central carbon metabolism proposed by Han et al.^10^ proposed that arginine could bypass the bottleneck of glutamate dehydrogenase and enter the TCA cycle via the urea cycle. Arginine is part of the urea cycle and can be rapidly cleaved to urea and ornithine by the arginase enzyme (Car1). The ornithine is condensed by Arg3 with carbamyl phosphate and forms citrulline. This is then condensed by Arg1 with aspartate to form argininosuccinate which is then cleaved to fumarate—one of the TCA cycle intermediates. We also predict that urea, cleaved from arginine, can be hydrolysed into two carbon dioxide molecules and ammonia. This ammonia can feed into the *GLN1*/*GLT1* pathway and be converted into glutamate at the expense of ATP and NADH (Fig. 1). These pathways produce NAD^+^ which can be used to convert glutamate to α-ketoglutarate via NAD^+^-dependent *GDH2*. Indeed, we observed a similar NAD^+^ and NADH ratio between the *ghd3*/*gdh3* mutant and the wild-type when grown in arginine medium (Supplementary Fig. 6A). Lastly, additional genetic manipulations should be performed such as restoring the deleted *GDH* genes or overexpression *GDH* genes in the knockout mutants. If the phenotypes of the mutants can be successfully restored to wild type, this will confirm that only the correct genes have been deleted and further validate the specific phenotypes ascribed to each *GDH* mutant.

Lastly, the results of this study consistently indicated the importance of redox potential and nitrogen metabolism to the regulation of hyphal development. We observed differential morphogenetic responses between the *gdh3*/*gdh3* mutant and the wild-type strain when grown on proline as the sole carbon and nitrogen source. We propose that NADH-dependent glutamate dehydrogenase activity is favoured over NADPH production to meet the immediate energy requirements of the starved wild-type cells. The excess production of NADH during the catabolism of proline in the *gdh3*/*gdh3* mutant could be at the expense of NAD^+^ supply and formation of the NADPH needed for cellular division and hyphal development. On the other hand, arginine overcomes the disequilibrium of the cells’ redox balance through the bypass of glutamate dehydrogenase and enters the TCA cycle via the urea cycle. Further studies such as generating knockout mutations in other genes involved in maintaining the cell’s redox balance could also be undertaken to better characterise *C. albicans* morphogenesis in relation to central carbon metabolism. These studies could identify key enzymes involved in maintaining the cell’s redox balance. Such enzymes could be developed as targets which when inhibited would block the morphological transition in *C. albicans*—a major virulence factor for this opportunistic pathogen—and provide additional therapeutic options to overcome *C. albicans* infections.

## Methods

### *Candida albicans* strains and plasmids

*C. albicans* strain SC5314^22^ was used as the wild-type parental strain from which mutants were generated (Table 2). The *SAT1* flipper fragment containing the nourseothricin resistance gene was amplified from plasmid pSFS2^20^. Ethics approval to develop genetically modified *C. albicans* strain SC5314 was granted by the Environment Risk Management Authority of New Zealand (ER-AF-NO3P-3 14/11).

**Table 2:** *C. albicans* wild-type and mutant strains used in this study

Strains	Genotypes	References
SC5314	Wild-type	^22^
GDH2_M1	SC5314:: GDH2/gdh2Δ:::SAT1-FLP-FRT^a^	This study
GDH2_M2	SC5314:: GDH2/gdhΔ:::FRT^b^	This study
GDH2_M3	SC5314:: gdh2Δ:::FRT/gdh2Δ:::SAT1-FLP-FRT	This study
GDH2_M4	SC5314:: gdh2Δ:::FRT/gdh2Δ::FRT	This study
GDH3_M1	SC5314:: GDH3/gdh3Δ::SAT1-FLP-FRT	This study
GDH3_M2	SC5314:: GDH3/gdh3Δ::FRT	This study
GDH3_M3	SC5314:: gdh3Δ::FRT /gdh3Δ::SAT1-FLP-FRT	This study
GDH3_M4	SC5314:: gdh3Δ::FRT /gdh3Δ::FRT	This study

^a^SAT1-FLP-FRT denotes SAT1 flipper cassette

^b^FRT is the abbreviation for FLP recombination target

### Chemicals and reagents

All chemicals used in this study were of analytical grade. Methanol, chloroform, sodium bicarbonate, and sodium hydroxide were obtained from MERK (Damstadt, Germany). The internal standard 2,3,3,3-d_4_-alanine, the derivatisation reagent methyl chloroformate (MCF), and pyridine were purchased from Sigma-Aldrich (St. Louis, USA). Anhydrous sodium sulphate and farnesol were obtained from Fluka (Steinheim, Germany). U-^13^C5 l-proline and U-^13^C6 arginine were acquired from Cambridge Isotope Laboratory Inc. (Tewksbury, USA). Two types of DNA polymerases were utilised in this study: KOD hot start DNA polymerase from NovgenR (Darmstadt, Germany), used to construct the *SAT1* flipper disruption cassette, and TaKaRa Ex TaqTM from Takara Bio Inc. (Shiga, Japan), used for colony PCR amplifications. The antibiotic nourseothricin was purchased from WERNER BioAgents (Jena, Germany). The QIAquick Gel Extraction kit was supplied by Qiagen (Valencia, CA, USA) and the Alkali-cation yeast transformation kit was obtained from Qbiogene (Heidelberg, Germany).

### Primers designed for this study

Primers were designed using Oligo 6 software (MBI, USA) and synthesised by either Invitrogen^TM^ (Victoria, Australia) or IDT (Leuven, Belgium). A list of DNA primers used in this study is presented in Supplementary Table 1.

### Construction of SAT1 flipper disruption cassette

Both alleles of target genes were deleted from *C. albicans* SC5314 using the *SAT1* flipper method^20^. Briefly, ~0.4 kb DNA upstream (5′ flank) of the open reading frame (ORF) was amplified by PCR of genomic DNA using primers P1 and P2 (Supplementary Table 1 and Supplementary Fig. 1). Similarly, ~0.4 kb DNA downstream (3′ flank) of the open reading frame (ORF) was amplified by PCR of genomic DNA using primers P3 and P4. The *SAT1* flipper fragment was amplified from plasmid pSFS2^20^ using primers P5 and P6 that contained 5′ extensions, which were complementary to the 5′ ends of primers P2 and P3, respectively. Lastly, all three purified DNA fragments were joined together by fusion PCR^23^ using primers P1 and P4 to complete the construction of *SAT1* flipper disruption cassette. The *SAT1* flipper disruption cassette was isolated and gel-purified using the QIAquick Gel Extraction kit (Qbiogene). In brief, the band containing the DNA cassette was excised from the agarose gel and incubated with binding buffer at 50 °C for 10 min. After complete dissolution of the gel, the sample was added to a Qiagen spin column which was then centrifuged at 9200 × *g* for 1 min. The spin column was washed with 0.75 mL of washing buffer followed by centrifugation at 9200 × *g* for 1 min, twice. The DNA cassette was eluted from the spin column by adding 50 µL of elution buffer and the column was centrifuged at 15,700 × *g* for 1 min.

### *C. albicans* transformation and selection of transformants

*C. albicans* strain SC5314 was transformed by a combination of heat shock and lithium/caesium acetate provided by the Alkali-cation yeast transformation kit (Qbiogene). The transformation protocol was adapted from the kit instructions and the method described by Hernday et al.^24^. A colony of *C. albicans* was used to inoculate 10 mL of YPD medium (6 gL^−1^ yeast extract, 3 gL^−1^ peptone, and 10 gL^−1^ glucose at pH 6.5) in shake flasks which was incubated overnight at 30 °C in a rotary shaker. This culture was then diluted into 30 mL of YPD medium to give an initial OD_600_ of 1.0 and incubated at 30 °C in the rotary shaker for 4 h. After centrifugation at 15,700 × *g* for 5 min the supernatant was discarded and the cell pellet was repeatedly washed in 5 mL of TE buffer and resuspended in 3 mL of lithium/caesium acetate solution (Qbiogene). The cell suspension was incubated for a further 30 min at 30 °C with shaking. The cells were then isolated by centrifugation and resuspended in 300 µL of TE buffer. The following solutions were mixed together: 100 µL of cells in TE buffer, 10 µL of herring sperm DNA (10 mg mL^−1^), 10 µL of *SAT1* flipper disruption cassette (0.1 µg µL^−1^), and 5 µL of histamine solution (Qbiogene). The mixture was incubated at room temperature for 15 min, followed by adding 1 mL of diluted PEG (0.8 mL of PEG + 0.2 mL of TE/Cation – Kit components) and incubated at room temperature for another 3 h. After this, the cells were exposed to heat shock at 42 °C for 1 h. The cells were isolated by centrifugation and allowed to recover in 5 mL of YPD broth at 30 °C in a rotary shaker for 5 h prior to selective growth. Yeast cells were spread on YPD agar plates (YPD medium with 15 gL^−1^ agar) containing nourseothricin (200 µg mL^−1^) and incubated at 30 °C for 3 days. Nourseothricin-resistant colonies that contained correct 5′ and 3′ junctions in the disrupted alleles were confirmed by colony PCR using primers C1, C2, C3, and C4 (Supplementary Fig. 1 and Table 2).

The excision of the *SAT1* flipper cassette was induced by inoculating 5 mL of YPD broth, containing 2% maltose (without nourseothricin), with the nourseothricin-resistant strains and incubating them in a rotary shaker at 30 °C overnight. Approximately 100 cells were plated on YPD agar containing nourseothricin (25 µg mL^−1^) and incubated at 30 °C for 2 days. Slow growing colonies were patched onto YPD agar plates with and without nourseothricin (200 µg mL^−1^) to screen for nourseothricin-sensitive colonies. The excision of the *SAT1* flipper cassette was also confirmed by colony PCR using primers C1 and C4. The entire transformation procedure was repeated to delete both copies of *GDH* alleles. Lastly, a final PCR verification using primers C5 and C6 (Supplementary Fig. 1 and Table 2) was performed to confirm that no copies of the *GDH* alleles or the *SAT1* flipper disruption cassette were remaining in *C. albicans gdh*/*gdh* homozygous mutants.

### Morphology of mutant and wild-type strains

*C. albicans* strains were maintained on YPD agar plates at 30 °C. These cells were used to inoculate 100 mL of YPD broth which was incubated at 30 °C in a rotary shaker overnight. The cells were collected by centrifugation at 2000 × *g* for 5 min and washed in phosphate buffered saline (8 gL^−1^ NaCl, 0.2 gL^−1^ KCl, 1.44 gL^−1^ Na_2_PO_4_, 0.24 g L^−1^ KH_2_PO_4_, at pH 7.5). The cells were resuspended in the desired growth media to an initial optical density (OD_600_) of 0.1. To study the morphology of wild-type and mutants strains under various growth conditions, the strains were cultured in 11 different growth media which, apart from the control MM medium, have been reported by other workers to stimulate hyphal formation in *C. albicans*. The compositions of these media are listed in Supplementary Table 2. The MM^−^ medium was the basic minimum mineral medium without any carbon or nitrogen sources. Other hyphae-inducing media were MM^−^ medium supplemented with l-arginine (10 mM), l-glutamate (10 mM), l-glutamine (10 mM), l-proline (10 mM), or *N*-acetylglucosamine (2.5 mM) at pH 6.5. The effect of ammonium (10 mM) or urea (10 mM) as the only nitrogen source on the growth of the different *C. albicans* strains at pH 6.5 was also investigated. The inoculated media were incubated in a rotary shaker at 37 °C for 3 h. The morphology of *C. albicans* cells in each growth medium was observed using a phase contrast microscope (DMR, Lecia).

### Growth characterisation and growth rates

*C. albicans* mutant and wild-type strains were cultured in 30 mL of minimum mineral (MM) or MM^−^ media supplemented with either l-arginine or l-proline. The growth yield of each culture was monitored by dry-weight measurements each hour until the stationary growth phase was reached. The growth rate was calculated by Eq. 1^25^, in which *µ* refers to the exponential growth rate; *B*_0_ denotes biomass measured at the beginning of the exponential growth; *B* is the biomass measured at the end of exponential growth; *t*_o_ is the time (h) at the beginning of exponential growth; and *t* is the time (h) at the end of exponential growth.

1 $$\mu = \frac{{2.303 \times ({\mathrm{log}}({\mathrm{B}}) - {\mathrm{log}}({\mathrm{B}}_0))}}{{t - t_0}}$$

The growth rate formula

### Growth of yeast for metabolomic analysis

*C. albicans* wild-type and mutant strains were cultured in 200 mL of YPD broth at 30 °C in a rotary shaker overnight. The cells were collected by centrifugation at 2000 *g* for 5 min and washed in phosphate buffered saline. The cells were resuspended in 30 mL of culture media to an initial OD_600_ of 0.2 and incubated in a rotary shaker at 37 °C. There were 5 different culture media used in metabolomics and isotope labelling experiments: (1) Minimum mineral medium (MM medium) at pH 6.5; (2) Arginine medium: MM^−^ medium supplemented with l-arginine (10 mM) at pH 6.5; (3) ^13^C_6_-arginine medium: MM^−^ medium supplemented with 30% ^13^C_6_-labelled (30% ^13^C_6_ and 70% ^12^C_6_) l-arginine (10 mM) at pH 6.5; (4) Proline medium: MM^−^ medium supplemented with l-proline (10 mM) at pH 6.5; (5) ^13^C_5_-proline medium: MM^−^ medium supplemented with 30% ^13^C_5_-labelled (30% ^13^C_5_ and 70% ^12^C_5_) l-proline (10 mM) at pH 6.5.

### Sampling and quenching of microbial metabolism

Five culture flasks (30 mL) of each *C. albicans* strain growing on MM medium, ^13^C_6_-arginine medium, or ^13^C_5_-proline medium were harvested at late exponential growth phase, while one flask of each culture growing on non-isotope labelled medium was harvested to estimate the natural abundance of ^13^C in the samples. A portion (5 mL) of each culture was centrifuged at 2000 ⨯ *g* for 5 min to remove *C. albicans* cells, and the supernatants were used for the analysis of extracellular metabolites. Another small portion (2 mL) of each culture was sampled and stored at −80 °C for ATP analysis. The remaining 23 mL of each culture was quickly filtered under vacuum (Air Cadet vacuum/pressure station, Thermo), immediately washed with cold saline solution (9.5 g L^−1^ NaCl at 1–2 °C) and quenched in cold methanol/water (1:1 v/v) at −30 °C as described by Smart et al.^26^.

### Sample preparation for metabolite analysis

The internal standard 2,3,3,3-d_4_-alanine (0.3 μmol/sample) was added to each sample before extraction. Freeze-thaw cycles and cold methanol/water were used to extract the intracellular metabolites from the quenched cell pellets according to protocols described by Smart et al.^26^. The intracellular metabolite extracts and 2 mL of spent culture medium containing extracellular metabolites were freeze-dried (BenchTop K manifold freeze dryer, VirTis) prior chemical derivatisation.

### Chemical derivatisation of metabolites

All freeze-dried samples were derivatised using the MCF method described by Smart et al.^26^. In brief, the freeze-dried samples were resuspended in 200 μL sodium hydroxide (1 M) and transferred to a silanised glass tube, then mixed with 167 µL methanol and 34 µL pyridine. The derivatisation was initiated by adding 20 µL MCF followed by vigorously mixing for 30 s, and then a further 20 µL MCF was added followed by vigorously mixing for 30 s. To separate MCF derivatives from the reaction mixture, 400 µL chloroform was added and vigorously mixed for 10 s followed by the addition of 400 µL sodium bicarbonate (50 mM), and mixing for an additional 10 s. The aqueous layer was discarded and the remaining water was removed with anhydrous sodium sulphate before samples were transferred to GC-MS vials.

### Gas chromatography-mass spectrometry (GC-MS) analysis

The MCF derivatives were analysed in an Agilent GC7890 system coupled to a MSD5975 mass selective detector (EI) operating at 70 eV. The gas column used for all analyses was a ZB-1701 GC capillary column (30 m ⨯ 250 μm id ⨯ 0.15 μm with 5 m guard column, Phenomenex). The analysis parameters were set according to Smart et al.^26^. Samples were injected under pulsed splitless mode with the injector temperature at 290 °C. The helium gas flow through the GC-column was set at 1 mL min^−1^. The interface temperature was set to 250 °C and the quadrupole temperature was 200 °C. The mass spectrometry was operated in scan mode and started after 5.5 min with the mass range between 38 and 550 atomic mass units (amu) at 2.85 scans s^−1^.

### Biomass quantification

The cell debris collected after intracellular metabolite extraction was dried using a domestic microwave (250 W for 20 min) and weighed in order to estimate the total biomass content (dry weight) of each sample.

### Data mining, data normalisation, and data analysis

AMDIS software was employed to deconvolute GC-MS chromatograms and identify metabolites using our in-house MCF mass spectra library. The identifications were based on both MS spectrum of the derivatised metabolite and its respective chromatographic retention time. The relative abundance of identified metabolites was determined by ChemStation (Agilent) by using the GC base-peak value of a selected reference ion. These values were normalised by the biomass content in each sample as well as by the abundance of internal standard (2,3,3,3-d_4_-alanine). A univariate analysis of variance (ANOVA) was used to determine whether the relative abundance of each identified metabolite was significantly different between wild-type and mutant strains under each growth condition. Our Pathway Activity Profiling (PAPi) algorithm^27^ was used to predict and compare the relative activity of different metabolic pathways between different *C. albicans* strains under the growth conditions tested. This programme is linked to the KEGG online database (http://www.kegg.com) and uses the number of metabolites identified from each pathway and their relative abundances to predict which metabolic pathway is likely to be active in the cell. The entire data mining, data normalisation, and pathway activity predictions were automated in our in-house R software package as described in Smart et al.^26^. Graphical representations of the results were produced by ggplot2 R packages^28^.

### Calculating ^13^C-labelling enrichment in the detected metabolites

The distribution of ^13^C-labelling from arginine or proline in the identified metabolite pool was determined by calculating the ratio of ^13^C to ^12^C in the major mass fragments. ^13^C-labelled metabolites were first identified based on their chromatographic retention time obtained from AMDIS. Together with the electron-impact fragmentation pattern of each identified MCF derivatised ^13^C-labelled metabolite, the degree of labelling was estimated based on the variation observed in the fragmentation pattern of ^13^C-labelled metabolites in comparison to their counterpart ^12^C mass spectrum. Lastly, the total percentage of ^13^C labelling was subtracted from the natural abundances of ^13^C calculated from samples without added labelled substrates (Equation 2). This enabled identification of ^13^C-enrichment in the metabolites originating from the catabolism of ^13^C-labelled arginine and proline.

2 $$\begin{array}{l}{\mathrm{\% }}\,of\,isotope\,labelling\,enrichment = Enrichment\,^{13}C\left( {\frac{{\left( {M \,+\, n} \right)\,ion\,mass\,height}}{{\left( M \right)\,ion\,mass\,height}} \times 100} \right)\\ \,\,\,\,\,\,\,\,\,\,\,\,\,\,\,\,\,\,\,\,\,\,\,\,\,\,\,\,\,\,\,\,\,\,\,\,\,\,\,\,\,\,\,\,\,\,\,\,\,\,\,\,\,\,\,\,\,\,\,\,\,\,\,\,\,\,\,\,\,\,\,\,\,\,\,\,\,\,\,\,\,\, -\, Natural\,^{13}C\left( {\frac{{\left( {M \,+\, n} \right)ion\,mass\,height}}{{\left( M \right)\,ion\,mass\,height}} \times 100} \right)\end{array}$$

The percentage of isotope labelling enrichment formula. M is the ^12^C ion of the metabolite; *n* is a number of m/z higher than M; M + *n* is the ^13^C ion of the metabolite; Enrichment ^13^C is ^13^C-labelled metabolites extracted from the cells grown in isotope enrichment medium; and Natural ^13^C is the natural abundance of ^13^C found in the cells grown in non-isotope enriched medium.

### Quantification of intracellular ATP pools

The intracellular pool of ATP was determined by using the luminescence ATP detection assay ATPlite^TM^ (PerKinElmer). This assay measures light emission caused by the enzymatic reaction between ATP and luciferin-luciferase. Medium without cells was used to determine the assay background signal for each medium. Three technical replicates of 100 μL culture were taken from each culture flask sample prior to quenching cells in cold methanol/water. The cells were lysed and the total ATP content was quantified according to the manufacturer’s instructions using ATP standard curves obtained from medium spiked with ATP standard solutions (one calibration curve for each medium). Light emission was measured using a bioluminescence plate reader (Envison^TM^, PerkinElmer).

### Quantification of intracellular NAD^+^/NADH and NADP^+^/NADPH pools

The intracellular pools of NAD^+^/NADH and NADP^+^/NADPH were quantified by using the Fluorimetric Amplite^TM^ NAD^+^/NADH and NADP^+^/NADPH assays (AAT Bioquest®, Inc.). This assay measures light of wavelength of 590 nm emitted by an enzymatic reaction between fluorescent enzymes and cofactors when exposed to light with a wavelength of 540 nm. The cells in three technical replicates of 1.25 ml culture were collected and washed with PBS buffer (pH 7.4). The cells were lysed in cell lysis buffer with a TissueLyser (Qiagen) at 30 Hz for 5 min. The cofactors from cell lysates were measured according to the manufacturer instruction using cofactor standard curves. Fluorescence was measured using a fluorescence plate reader (Envison^TM^, PerkinElmer) at Ex/Em = 540/590 nm.

### Reporting Summary

Further information on experimental design is available in the Nature Research Reporting Summary linked to this article.

## Supplementary information

Supplementary Figures

Reporting Summary

## Data Availability

The data that support the findings of this study are available from the corresponding author upon reasonable request.

## Supplementary information

**Supplementary information** accompanies the paper on the *npj Biofilms and Microbiomes* website (10.1038/s41522-019-0086-5).
