# Supplementary material for: A metabolomic study of the effect of Candida albicans glutamate dehydrogenase deletion on growth and morphogenesis
Source: NPJ Biofilms Microbiomes. 2019 Apr 8;5:13. doi: 10.1038/s41522-019-0086-5 (PMC6453907; doi:10.1038/s41522-019-0086-5)
Supplement: Supplementary file 1 — Supplementary Figures [file 41522_2019_86_MOESM1_ESM.pdf]

Supplementary Table 1. DNA primers used in this study

Primer Name	Sequence (5’ to 3’)
GDH2_P1	CCCACCTTTTTTTTTCCAC
GDH2_P2	GATGTAGTGTTTGATGTGGTTC
GDH2_P3	ATTCCTTGTAAGTTTCTCTGTG
GDH2_P4	TCGCAAAGGAAAATCAAGT
SAT1/GDH2_P5	GAACCACATCAAACACTACATCAGGAAACAGCTATGACCATG
SAT1/GDH2_P6	CACAGAGAAACTTACAAGGAATCAGTCACGACGTTGTAAAAC
GDH2_C1	TTGGTTGTCTTCCTTCTTTC
GDH2_C2	GCTTTTGTTCCCTTTAGTGA
GDH2_C3	TTGGCATACGATTAGAGACAC
GDH2_C4	GAGGGTAGTTTTCACAATTTC
GDH2_C5	TTACAGAACCACATCAAACACT
GDH2_C6	GGCAATGAATCCAACAATAC
GDH3_P1	TAGGCTTCGACAGTTCTGAC
GDH3_P2	ATTCTGGTTCGTGAGGTAAG
GDH3_P3	AAAGGGTATAAACGACATTCC
GDH3_P4	CTTTCCCTGCACTCTCTCAC
SAT1/GDH3_P5	CTTACCTCACGAACCAGAATAGGAAACAGCTATGACCATG
SAT1/GDH3_P6	GGAATGTCGTTTATACCCTTTCAGTCACGACGTTGTAAAAC
GDH3_C1	CTTCGCAATAGTTAACCGTAC
GDH3_C2	GCTTTTGTTCCCTTTAGTGA
GDH3_C3	TTGGCATACGATTAGAGACAC
GDH3_C4	AAGACAACAACAACAATACACG
GDH3_C5	ACGGTTTCAGAGTTCAATACA
GDH3_C6	GCAATGTTGGCACCTTTC

Supplementary Table 2. Media used to induce morphogenesis in the *C. albicans* strains

Culture media	Media compositions	References
Minimum mineral medium (MM)[1]	D-glucose (10 g.L^{-1}), $(NHR_4R)R_2RSOR_4$ R(5 g.L^{-1}), $MgSOR_4R·7H_2O$ (0.5 g. L^{-1}), KH_2PO4 (6 g.L^{-1}), vitamins and trace metals at pH 6.5.	Modified from Verduyn, et al. [1]
Basic mineral medium (MM^-)	$MgSO_4·7H_2O$ (0.5 g.L^{-1}), KH_2PO_4 (3 g.L^{-1}), vitamins and trace metals at pH 6.5.	
Minimal-proline and *N*-acetylglucosamine medium (MPA)	MM^- medium supplemented with L-proline (10 mM) and *N*-acetylglucosamine (2.5 mM) at pH 6.5.	Modified from Sullivan and Shepherd [2]
Serum medium	MM^- medium supplemented with bovine serum (10 % $_{v/v}$) at pH 6.5.	Modified from Reynolds and Braude [3]
Lee's medium	$(NH_4)_2SO_4$ (5 g. L^{-1}), $MgSO_4·7H_2O$ (0.2 g.L^{-1}), KH_2PO_4 (2.5 g.L^{-1}), NaCl (5.0 g.L^{-1}), L-alanine(0.5 g.L^{-1}), L-leucine (1.3 g.L^{-1}), L-lysine (1.0 g.L^{-1}), L-methionine (0.1 g.L^{-1}), L-phenylalanine (0.5 g.L^{-1}), L-proline (0.5 g.L^{-1}), L-threonine (0.5 g.L^{-1}) , D-glucose (12.5 g.L^{-1}), and biotin (0.001 g.L^{-1}) at pH 6.5.	Lee, et al. [4]
MM^- + L-proline[1]	MM^- medium supplemented with L-proline (10 mM) at pH 6.5	Modified from Dabrowa, et al. [5]
MM^- + L-arginine[2]	MM^- medium supplemented with L-arginine (10 mM) at pH 6.5	Modified from Ghosh, et al. [6]
MM^- + *N*-acetylglucosamine	MM^- medium supplemented with *N*-acetylglucosamine (2.5 mM) at pH 6.5	Modified from Sullivan and Shepherd [2]
MM^- + L-glutamine	MM^- medium supplemented with L-glutamine (10 mM) at pH 6.5	
MM^- + L-glutamate	MM^- medium supplemented with L-glutamate (10 mM) at pH 6.5	
MM^- + D-glucose + ammonium	MM^- medium with D-glucose (10 g.L^{-1}), and supplemented with ammonium (10 mM) at pH 6.5	Modified from Holmes and Shepherd [7]
MM^- + D-glucose + urea	MM- medium with D-glucose (10 g.L^{-1}), and supplemented with urea (10 mM) at pH 6.5	

MM^- is the minimum mineral medium without any carbon or nitrogen sources. [1]Only these media were used in metabolomic analyses.

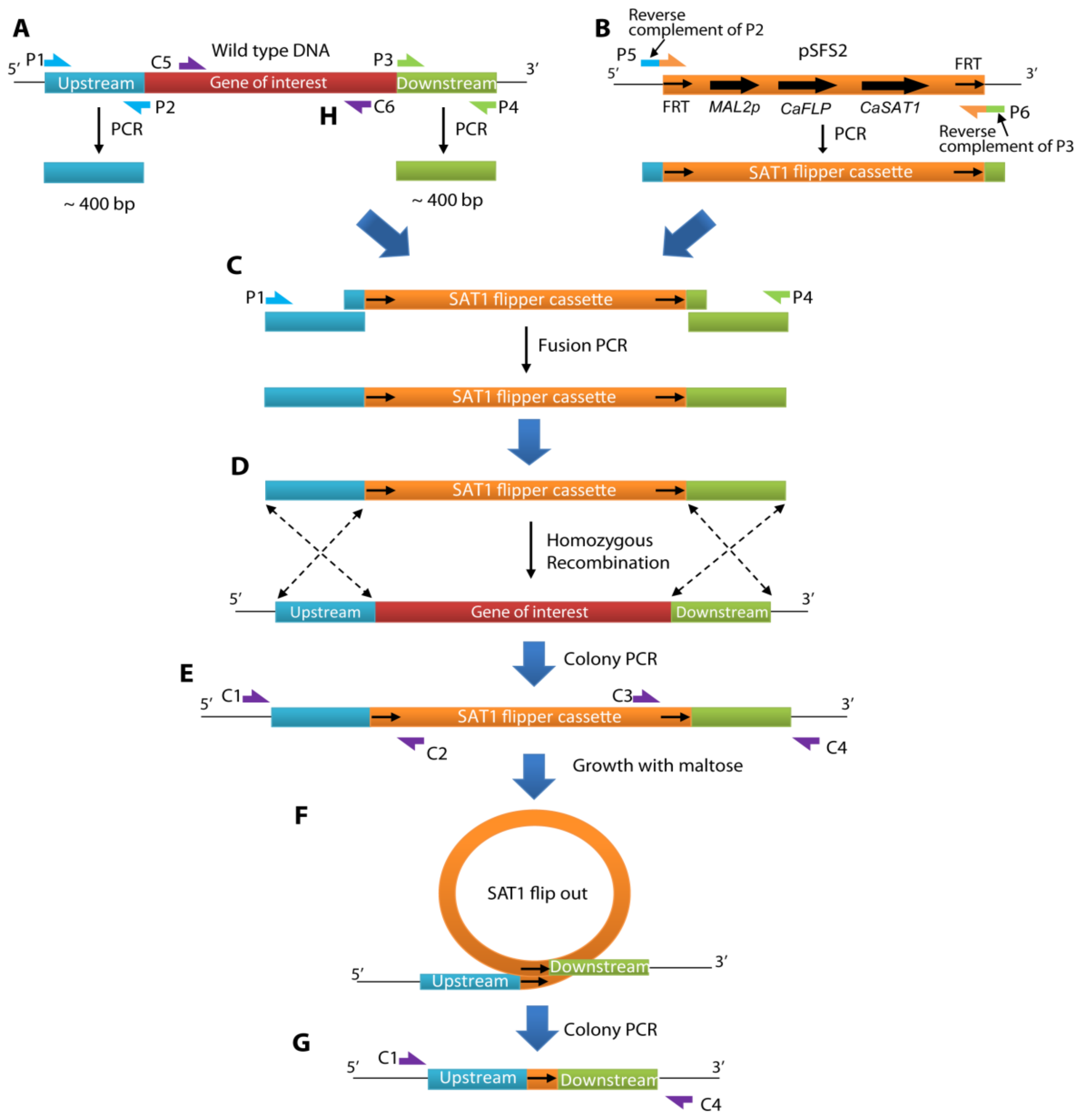

Supplementary Figure 2. The construction of *C. albicans* mutant strains. (A) PCR amplification of DNA fragments (~500 bp) upstream and downstream of target genes using primers P1, P2, P3, and P4. (B) PCR amplification of the *SAT1* flipper fragment from plasmid pSFS2 using primers P5 and P6. The *SAT1* flipper fragment consists of the maltose-activated promoter (MAL2p), *C. albicans*-adapted *FLP* gene (*CaFLP*), nourseothricin resistance gene (*CaSAT1*), and FLP recombination target sequence (FRT). (C) Construction of *SAT1* flipper disruption cassette by fusion PCR using primers P1 and P4. (D) Transformation of *C. albicans* with disruption cassettes by lithium acetate and gene disruption via homozygous recombination. (E) Verification of the correct integration of the cassette by PCR amplification of the 3’ and 5’ ends of disrupted alleles using primers C1, C2, C3, and C4. (F) Induction of excision of the *SAT1* flipper with maltose via *FLP*-mediate recombination between FRT sequences. (G) Confirmation of the excision of the *SAT1* flipper using PCR with primers C1 and C4. Lastly, primers C5 and C6 were used to confirm disruption of *GDH2* or *GDH3* alleles by colony PCR. The primer sequences are listed in **Table 2**.

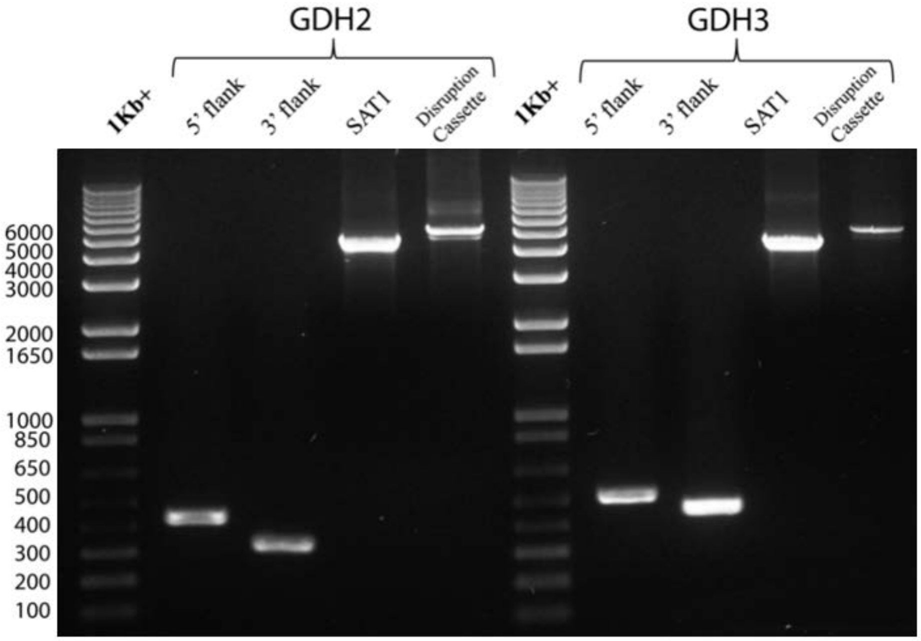

Supplementary Figure 1. Amplification of *SAT1* flipper disruption cassettes. The sequences 5' and 3' to the *GDH2* and *GDH3* open reading frames, and *SAT1* fragments were amplified by PCR and the disruption cassettes were generated by joining the three fragments using fusion PCR. All agarose gel results were derived from the same experiment and were processed in parallel.

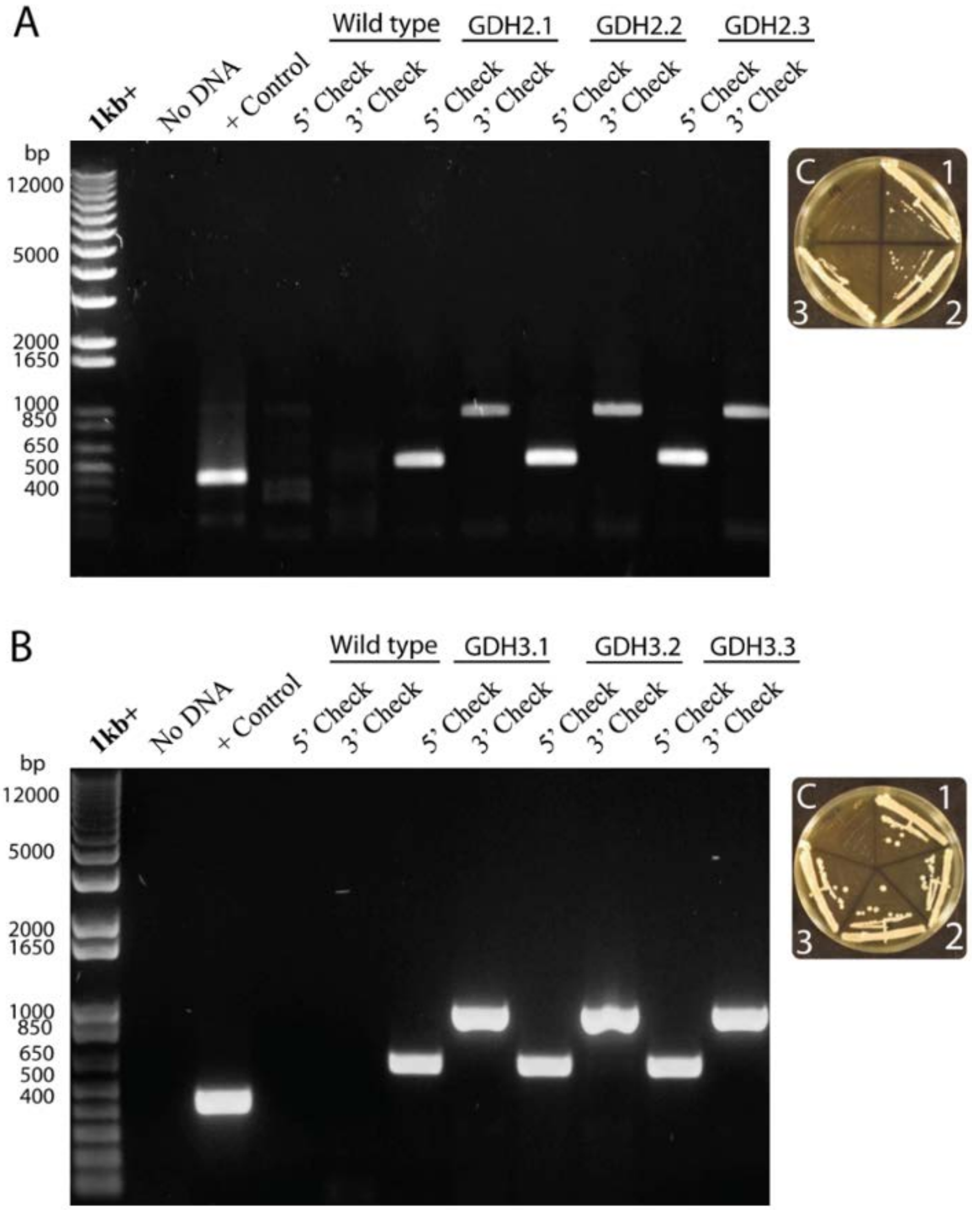

Supplementary Figure 3. Correct integration of *SAT1* flipper disruption cassettes. The 5' and 3' junctions of disrupted *GDH2* (A) and *GDH3* (B) alleles were verified by genomic PCR amplification. The nourseothricin-resistant strains were tested by growing cells on YPD agar containing 200 µg.mL^{-1} nourseothricin (30°C for three days). (**C**) 1, 2 and 3 on the agar plate photos represent the parental wild type and three independent transformants respectively. Parental cells were used as the negative control. All agarose gel results were derived from the same experiment and were processed in parallel.

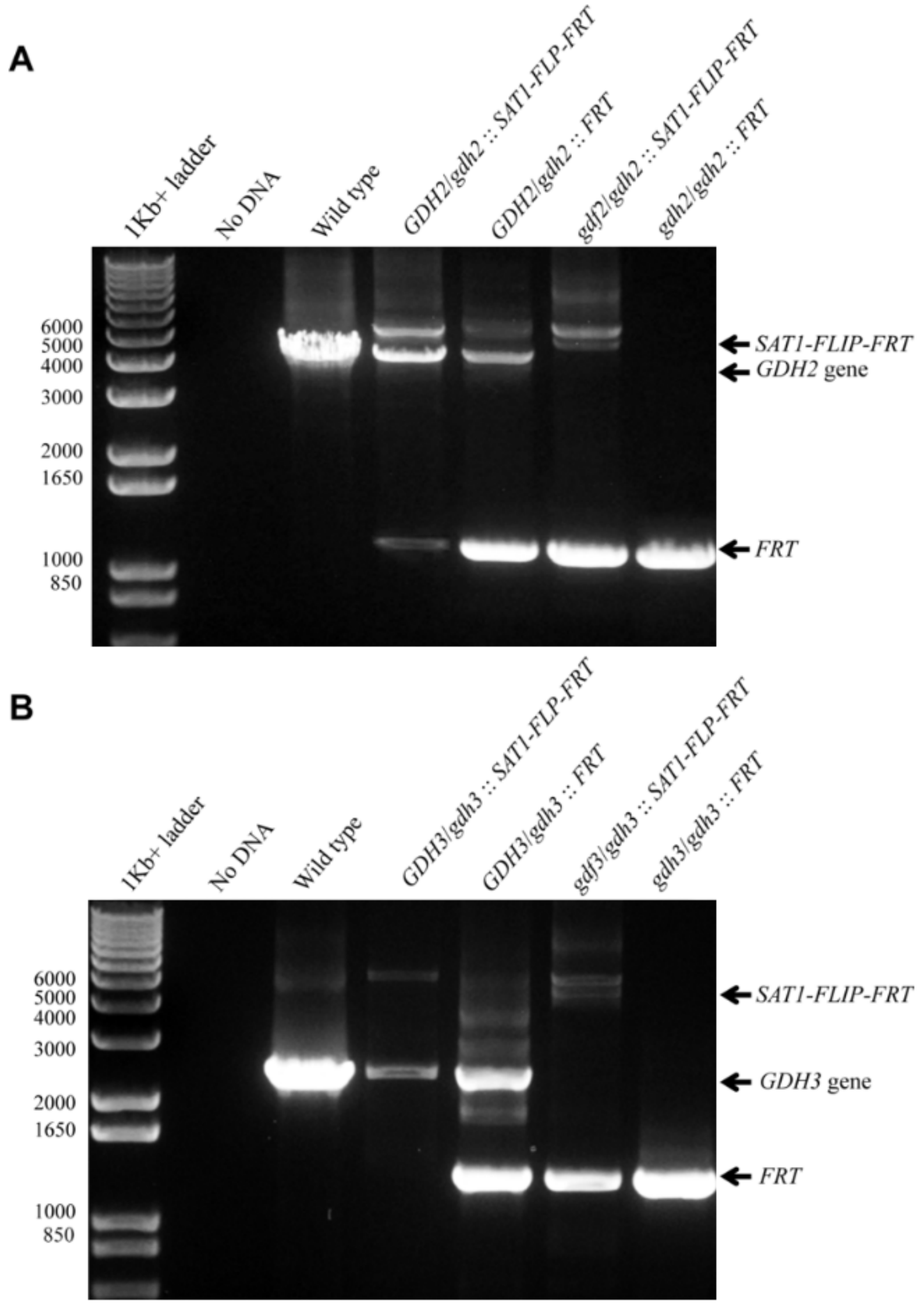

Supplementary Figure 4. Genomic PCR verification of gene disruption in different *C. albicans* strains constructed in this study using primers C1 and C4. (**A**) *C. albicans* strains associated with *GDH2* gene knockout. (**B**) *C. albicans* strains generated during *GDH3* gene knockout. The sizes of DNA fragments (in bp) are indicated on the left and the identities of the fragments are indicated on the right. *SAT1-FLP-FRT* is the *SAT1*-flipper cassette and *FRT* is the *FLP* recombination sequence, which remains in the cell after excision of the *SAT1* flipper cassette. All agarose gel results were derived from the same experiment and were processed in parallel.

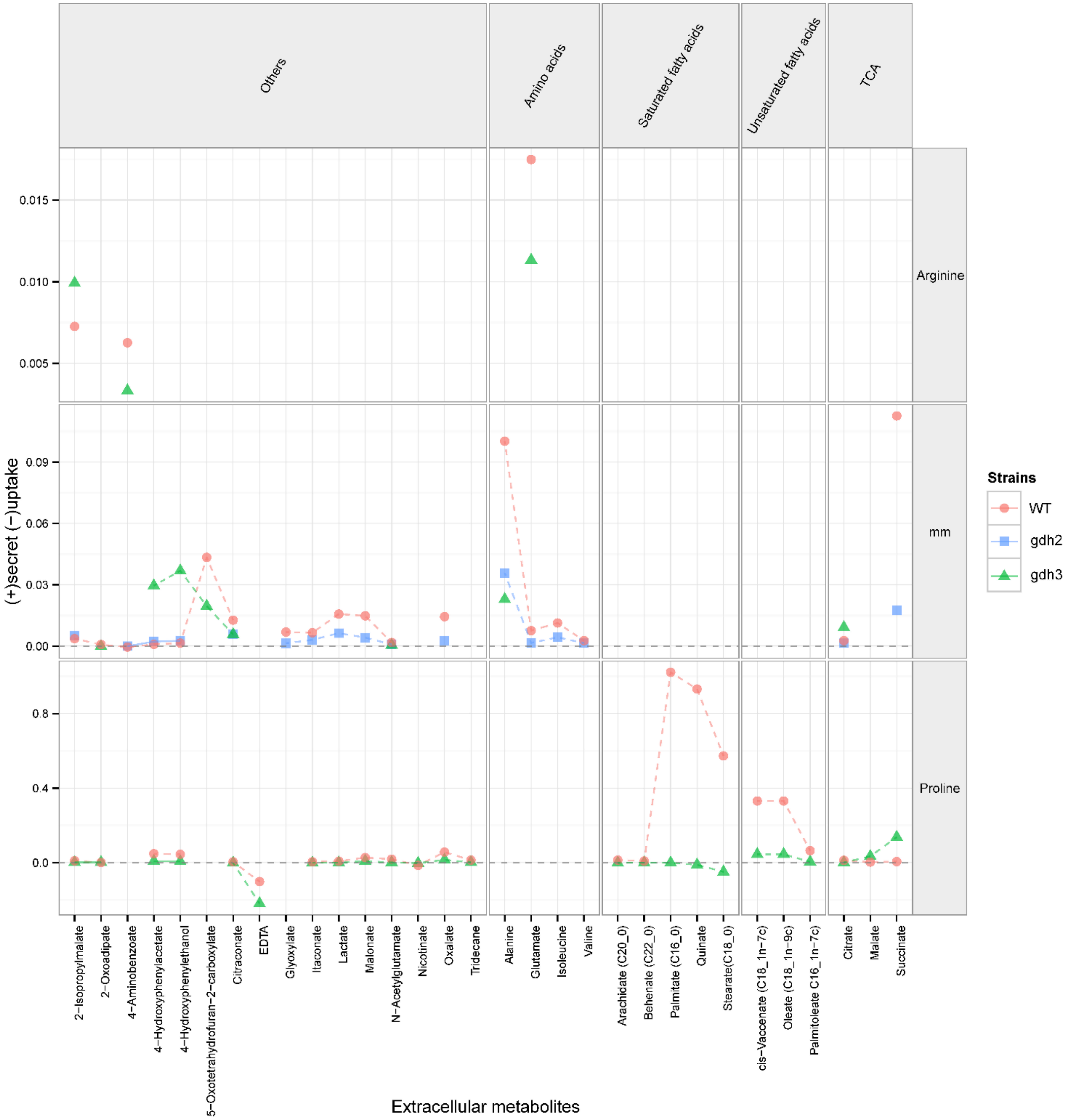

Supplementary Figure 6. The relative concentrations of extracellular metabolites for *C. albicans* mutants and wild type (WT) cells when cultured on arginine, proline, or minimum mineral (MM) media. The relative concentrations of identified metabolites were normalised to an internal standard (d_4-alanine) and biomass before the relative concentrations of the corresponding metabolites found in uninoculated culture medium were subtracted. The line y=0 distinguishes secretion of metabolites (positive values) from consumption of metabolites from the medium (negative values). Only the metabolites for which there was a statistically significant (Tukey's honest significance test, p-value < 0.05) difference in concentrations between wild type and mutant strains are displayed.

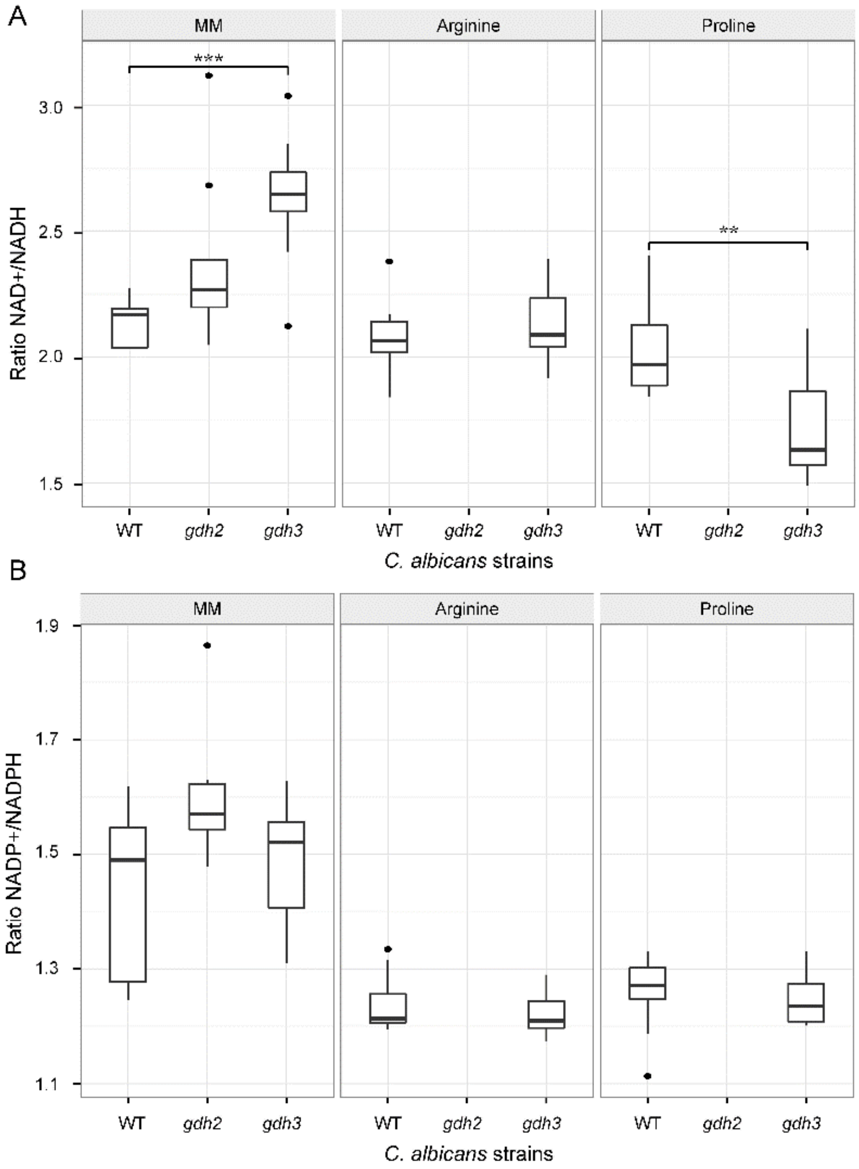

Supplementary Figure 7. Ratios of cofactors in the wild-type (WT), *gdh2*/*gdh2*, and *gdh3*/*gdh3* strains of *C. albicans* incubated in arginine, proline, or minimum mineral (MM) media. (**A**) The ratio of NAD^+/NADH in C. *albicans* mutant and wild-type strains. (**B**) The ratio of $NADP^+$/NADPH in *C. albicans* mutant and wildtype strains. There are no cofactor ratios for the *gdh2*/*gdh2* mutant incubated in arginine or proline media because this mutant was unable to grow under these conditions. The distributions of boxplot are minimum, 25th percentile, median, 75th percentile, and maximum (from bottom to upper direction). Black dots are outliers (>1.5 times of interquartile range). Nine experimental replicates were collected and measured from each group. Tukey's honest significance test ** (p-value< 0.05) and *** (p-value< 0.001).

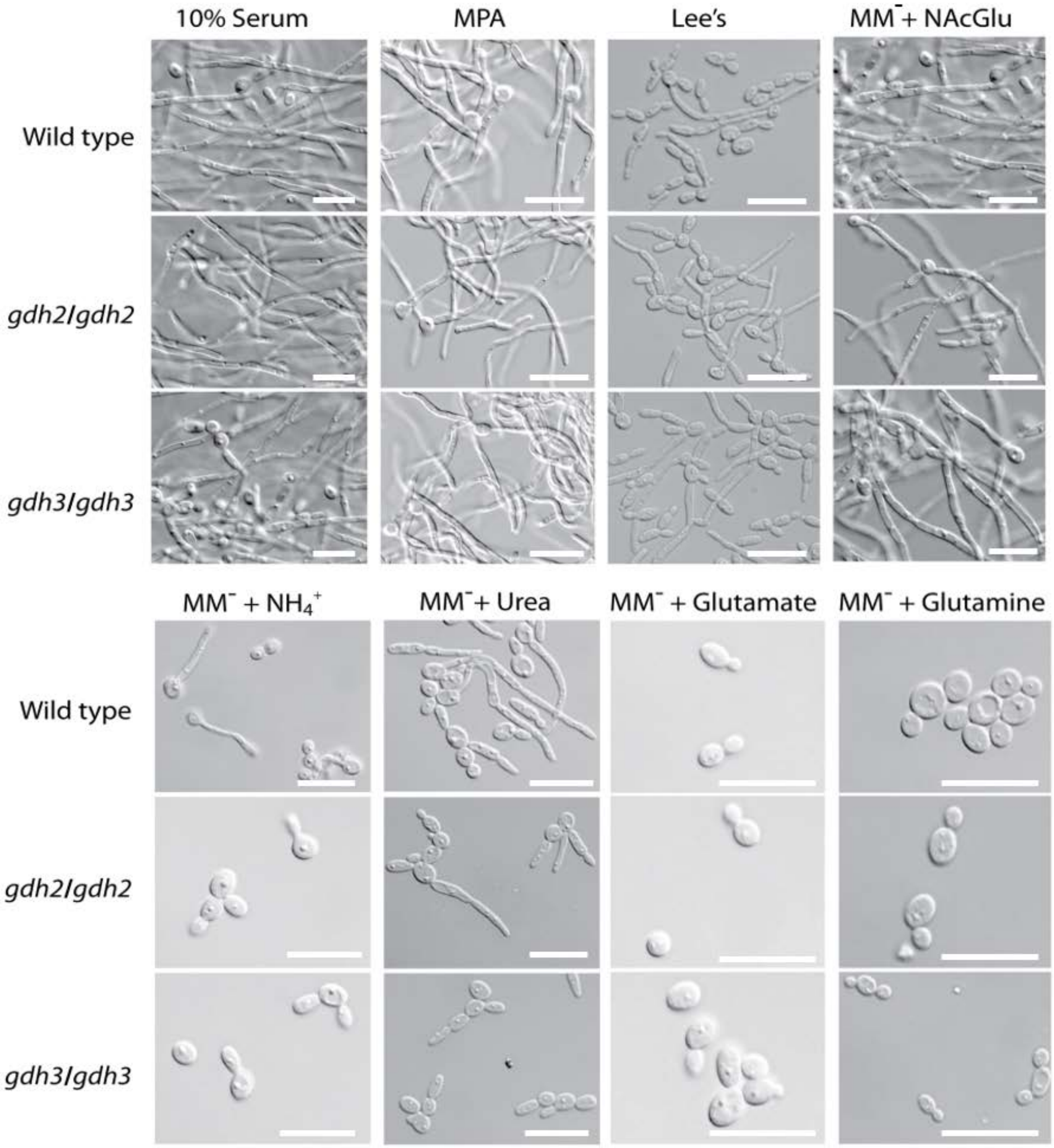

Supplementary Figure 5. The morphologies of *gdh2/gdh2*, *gdh3/gdh3*, and wild type strains of *C. albicans* grown under hyphae-inducing conditions (not shown in Figure 2). MM^- is the minimum mineral medium without any carbon or nitrogen source. MM^- medium was supplemented with either N-acetylglucosamine (NAcGlu), glutamate, or glutamine. When MM^- medium was supplemented with ammonium (NH_4^+) or urea the medium was also supplemented with 1 % glucose in. The wild type strain is *C. albicans* SC5314. The photos were taken by Nomarksi contrast microscopy with 100x magnification. The scale bars on each panel represent 10 μm.

References

1 Verduyn, C., Postma, E., Scheffers, W. A. & Van Dijken, J. P. Effect of benzoic acid on metabolic fluxes in yeasts: A continuous-culture study on the regulation of respiration and alcoholic fermentation. *Yeast* **8**, 501-517 (1992).

2 Sullivan, P. A. & Shepherd, M. G. Gratuitous induction by *N*-acetylmannosamine of germ tube formation and enzymes for *N*-acetylglucosamine utilization in *Candida albicans*. *Journal of Bacteriology* **151**, 1118-1122 (1982).

3 Reynolds, R. & Braude, A. I. The filament inducing property of blood for *Candida albicans*; its nature and significance. *Clinical Research Procedure* **4**, 40 (1956).

4 Lee, K. L., Buckley, H. R. & Campbell, C. C. An amino acid liquid synthetic medium for the development of mycelial and yeast forms of *Candida albicans*. *Sabouraudia Journal of Medical and Veterinary Mycology* **13**, 148-153 (1975).

5 Dabrowa, N., Taxer, S. S. S. & Howard, D. H. Germination of *Candida albicans* induced by proline. *Infect. Immun.* **13**, 830-835 (1976).

6 Ghosh, S. *et al.* Arginine-Induced germ tube formation in *Candida albicans* is essential for escape from murine macrophage line RAW 264.7. *Infect. Immun.* **77**, 1596-1605 (2009).

7 Holmes, A. R. & Shepherd, M. G. Proline-induced germ-tube formation in *Candida albicans*: Role of proline uptake and nitrogen metabolism. *Journal of General Microbiology* **133**, 3219-3228 (1987).
